# Transient Duplication-Dependent Divergence and Horizontal Transfer Underlie the Evolutionary Dynamics of Bacterial Cell–Cell Signaling

**DOI:** 10.1371/journal.pbio.2000330

**Published:** 2016-12-29

**Authors:** Eran Even-Tov, Shira Omer Bendori, Shaul Pollak, Avigdor Eldar

**Affiliations:** Department of Molecular Microbiology and Biotechnology, Faculty of Life Sciences, Tel-Aviv University, Tel-Aviv, Israel; Massachusetts Institute of Technology, United States of America

## Abstract

Evolutionary expansion of signaling pathway families often underlies the evolution of regulatory complexity. Expansion requires the acquisition of a novel homologous pathway and the diversification of pathway specificity. Acquisition can occur either vertically, by duplication, or through horizontal transfer, while divergence of specificity is thought to occur through a promiscuous protein intermediate. The way by which these mechanisms shape the evolution of rapidly diverging signaling families is unclear. Here, we examine this question using the highly diversified Rap-Phr cell–cell signaling system, which has undergone massive expansion in the genus *Bacillus*. To this end, genomic sequence analysis of >300 *Bacilli* genomes was combined with experimental analysis of the interaction of Rap receptors with Phr autoinducers and downstream targets. Rap-Phr expansion is shown to have occurred independently in multiple *Bacillus* lineages, with >80 different putative *rap-phr* alleles evolving in the *Bacillius subtilis* group alone. The specificity of many *rap-phr* alleles and the rapid gain and loss of Rap targets are experimentally demonstrated. Strikingly, both horizontal and vertical processes were shown to participate in this expansion, each with a distinct role. Horizontal gene transfer governs the acquisition of already diverged *rap-phr* alleles, while intralocus duplication and divergence of the *phr* gene create the promiscuous intermediate required for the divergence of Rap-Phr specificity. Our results suggest a novel role for transient gene duplication and divergence during evolutionary shifts in specificity.

## Author Summary

Many molecular pathways are found multiple times in a given organism, where they are often reutilized for different functions. Such expansion of a family of pathways requires two main evolutionary processes—acquisition of additional copies of the pathway's genes and divergence of interaction specificity to prevent cross-talk between pathways while preserving interactions within each copy of the pathway. In bacteria, acquisition can occur horizontally, by transfer between different lineages, or vertically, by duplication within the lineage. Interaction specificity is thought to diverge through a promiscuous intermediate component that prevents loss of interaction during the process. In this work, we study the mechanisms underlying the extreme expansion of the Rap-Phr cell–cell signaling family in the *Bacillus* genus. Specificity of Rap-Phr interaction is critical for guiding preferential action towards kin. We find that horizontal transfer and not duplication guides the acquisition of an already divergent Rap-Phr variant. Surprisingly, duplication still has a key role during expansion, as duplication and subsequent divergence of the signaling molecule gene provide the promiscuous intermediate state needed for divergence of specificity. We therefore identify two complementary roles for horizontal and vertical processes in the evolution of social bacterial pathways.

## Introduction

### The Roles of Pathway Acquisition and Diversification in the Expansion of Bacterial Signal Transduction Families

**Fig 1:**
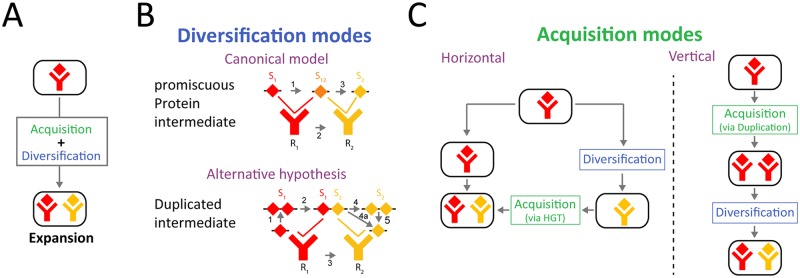
Evolutionary mechanisms for expansion of a pathway family. (A) Expansion of a signaling pathway family requires two processes—acquisition of another pathway and diversification of pathway specificity. (B) In the canonical model for divergence of protein–protein interaction specificity, it occurs through a promiscuous protein intermediate (Top). Mutations in one of the interacting proteins (arrow #1) form a promiscuous intermediate, which can interact both with the ancestral and evolved form of its partner (arrow #2). Subsequent mutations to the intermediate form narrow the specificity range to the novel form of the partner, completing the specificity shift (arrow #3) [12]. Alternatively, as we propose in this work, divergence can occur through a duplicated promiscuous state (Bottom). Duplication of one of the partners (arrow #1) and subsequent mutations in one of the duplicates (arrow #2) form an effective promiscuous form, where the ancestral copy interacts with the ancestral partner and the diverged copy with the evolved partner (arrow #3). Subsequent loss of the ancestral copy (arrow #4, 4a, and 5) completes the specificity shift. Note that the duplication needs to be transient for a full specificity shift to occur. (C) Acquisition of paralogous signaling pathways can occur in two forms: (left) horizontally, where pathway specificity diverges between lineages and then a divergent system is acquired by horizontal gene transfer (HGT), or (right) vertically, involving duplication and diversification within the lineage. Note that the order of acquisition and divergence is opposite in the two cases.

The evolution of signaling complexity often occurs by diversification and repeated utilization of signal transduction pathways [1–6]. This generally requires two processes: the acquisition of homologous copies of the pathway's components and the co-diversification of interacting components to ensure specificity of interaction within a pathway while avoiding cross-talk between pathways (Fig 1A) [7,8]. Bacteria have a multitude of signal transduction pathways, which have undergone evolutionary expansion and divergence of specificity, such as two-component systems [6,8–10], antisigma-sigma factors [11], and toxin-antitoxin systems [12,13]. The large number of available bacterial genomes allows for high-resolution analysis of evolutionary expansion, rendering bacterial signal transduction a favorable model system for studying diversification.

While eukaryotes can only acquire paralogous genes through duplications, bacteria can acquire them either by gene duplication (Fig 1C, right) or by horizontal transfer (Fig 1C, left) [14]. Previous works on the prevalence of these two processes in the acquisition of bacterial two-component signal transduction pathways have indicated that it is dominated by gene duplication, but it is also affected by horizontal transfer [15–17]. However, the coarse-grained resolution of these studies prevents the distinction between vertical acquisition and horizontal transfer between closely related strains [18,19].

The second requirement for paralogous expansion is the divergence of interaction specificity between pathways (Fig 1B). This is generally thought to evolve using a promiscuous form of one of the interacting components, which can interact with both variants of its partner (Fig 1B, top) [2,4,12,20–22]. The promiscuous form can be the ancestral state, subsequently evolving into two states of different specificity [4,20], or it can be an evolutionary intermediate between the ancestral specific state to a novel state [22,23]. The ability to distinguish between these two diversification scenarios typically depends on our capacity to infer and analyze the ancestral state from phylogenetic data [2,20,21]. A recent work used deep mutational scanning to show the abundance of promiscuous bacterial intermediates in the evolution of a bacterial toxin-antitoxin family [12]. This approach, however, cannot distinguish whether the promiscuous form is ancestral or intermediate or determine the evolutionary relevance of the identified diversifying trajectories. The modes by which rapidly diversifying signaling families expand are therefore still unclear.

### The Rap-Phr Quorum-Sensing Family as a Model for Diversification and Expansion of Bacterial Signal Transduction

The Rap-Phr cell–cell signaling system of *Bacilli* can serve as a model system to study bacterial modes of expansion and diversification [24–26]. The cytoplasmic Rap receptor can bind, and sometimes dephosphorylate, its target, leading to inhibition of target activity [27,28]. The cognate *phr* gene codes for a pre-polypeptide, which undergoes multiple cleavage events during its secretion, resulting in the release of a mature penta- or hexa-peptide Phr autoinducer [25,29–31]. The mature Phr peptide is transported into the cytoplasm through the oligopeptide permease system, where it can interact with Rap receptors [26], subsequently leading to major conformational changes in the Rap protein and preventing Rap from repressing its target [27,28,32,33].

Rap-Phr systems have mostly been studied in the *B*. *subtilis 168* lab strain. This strain encodes for eight paralogous *rap-phr* loci, each coding for a different Phr autoinducer. In addition, it encodes for three orphan *rap* genes that lack a cognate *phr* locus [33–35]. Despite the genomic expansion of paralogous Rap-Phr systems, they all have the same overall structural organization and most have a redundant function in repressing either Spo0F or ComA, two key response regulators of the *Bacillus* stress response network [31,36–40]. We recently demonstrated how social selection can explain the acquisition of additional Rap-Phr systems, despite their redundant regulation of the same target [41]. Some *rap-phr* loci are encoded by mobile genetic elements [31,39,42–47], and while many mobile-element-associated Rap systems maintain their repressive effect on Spo0F or ComA, some also play a direct role in controlling the mobility of their associated mobile genetic elements [44,47].

To study the expansion and diversification of the Rap-Phr family, we combined computational mining of available *Bacillus* genomes and experimental characterization of the target and autoinducer specificity of multiple Rap-Phr systems. We found that at the organismal level, acquisition of a novel Rap-Phr paralogous system occurred by horizontal gene transfer (Fig 1C, left). At the locus level, diversification of Rap-Phr specificity was facilitated by *phr* gene duplication or intragenic duplication of the Phr autoinducer coding sequence, followed by diversification of the autoinducer sequence. We show that the diverged duplicated *phr* form can serve as a promiscuous intermediate between two states of specificity (Fig 1B, bottom). Therefore, the extreme diversity of the Rap-Phr system results from a combination of horizontal and vertical processes operating at two different levels of genetic organization.

## Results

### Construction of a Bacilli Rap-Phr Database

To understand the extent to which Rap-Phr systems are prevalent in the *Bacillus* genus, we downloaded 413 whole-genome sequences of strains from this genus from the National Center for Biotechnology Information (NCBI) (S1 Data). The species association of these genomes is heavily biased towards genomes from the *B*. *subtilis* (127 strains) and *B*. *cereus* (216 strains) groups of species (S1 Fig). The conserved Rap structure, which includes a three-helix N-terminal and tetra-tricopeptide repeat C-terminal domain [24,32,33], allowed us to search for Rap homologs in all the genomes using the basic local alignment search tool for translated DNA (BLAST tblastn program). Following identification of Rap homologs, we searched for candidate *phr* genes, relying on the known organization of annotated phr genes (S2 Fig)—short open reading frames with a secretion signal sequence, located immediately downstream of the Rap gene in the same direction (see Methods) [24].

**Fig 2:**
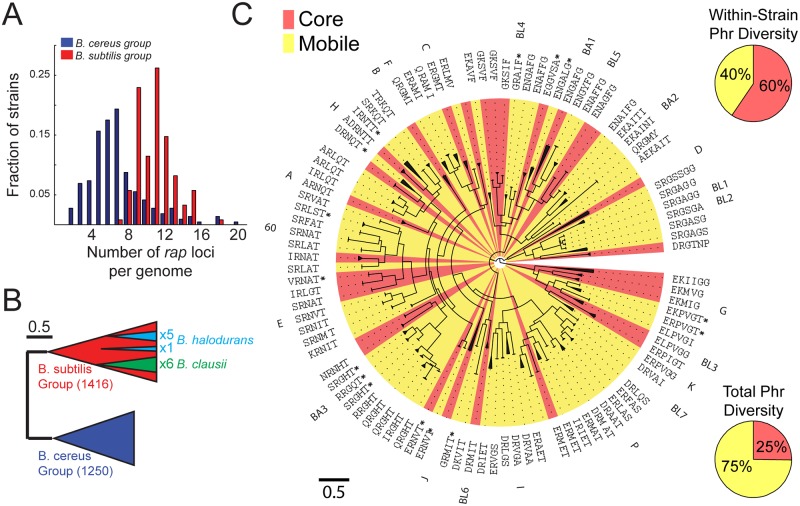
Diversity and accumulation of Rap-Phr systems in the *Bacillus* genus. (A) Distribution of the number of *rap* homologs in a given strain of the *B*. *subtilis* (red) and *B*. *cereus* (blue) groups (S5 Data). (B) A scheme of the phylogenetic tree of ~2,700 Rap proteins. The phylogeny is divided into two major clades, which coincide with the *B*. *subtilis* (red) and *B*. *cereus* (blue) groups. The six *rap* homologs of the single *B*. *clausii* isolate (green) are monophyletic and associated with the *B*. *subtilis* Rap clade. Five of the six Rap homologs of *B*. *halodurans* are monophyletic and originate from one node in the *B*. *subtilis* clade, whereas the sixth originates from a different node in this clade. The scale marks the length along tree equivalent to a 50% chance of amino-acid substitution per site. (C) Detailed phylogeny of ~1,500 Rap proteins of the *B*. *subtilis* group. The tree is clustered into monophyletic groups encoding for the same putative Phr peptide. Each cluster is marked with a triangle, the length of which is equal to the average distance of leaves in the subtree from the subtree base. The putative Phr peptides are listed next to each branch. The name of the characterized Rap from the cluster is also marked, if such exists. Background colors indicate whether the specific cluster is categorized as mobile (yellow) or core (red) based on guanine-cytosine (GC)-content (see text and S3 Fig). The scale marks the length along tree equivalent to a 50% chance of substitution per site. The top right pie chart shows the average fraction of mobile (yellow) and core (red) Rap-Phr systems encoded in an average *B*. *subtilis* group isolate. The bottom right pie chart shows the total fraction of mobile (yellow) and core (red) Rap-Phr systems in the *B*. *subtilis* group.

Our final database contained ~2,700 functional *rap* genes (S1 and S2 Data). *rap* genes were identified in all strains of the *B*. *subtilis* and *B*. *cereus* groups as well as in two evolutionarily distinct *Bacillus* species—*B*. *halodurans and B*. *clausii* (S1 Fig). Notably, all strains harboring at least one *rap* gene encoded for multiple *rap* paralogs (Fig 2A, S5 Data). The number of rap genes differed between groups, averaging 11 ± 2 (mean ± standard deviation [st.dev.]) in the *B*. *subtilis* group and 6 ± 3 in the *B*. *cereus* group. In *B*. *subtilis* 168, there are three orphan *rap* genes not accompanied by an adjoining *phr* gene, a scenario typical for *B*. *subtilis* strains, which have an average 2.7 ± 1 orphan raps per strain (mean ± st.dev.). In contrast, almost all (>95%) of the *rap* genes from the *B*. *cereus* group had an adjoining putative *phr* gene.

### Phylogenetic Analysis of Rap-Phr Diversity

We next performed a phylogenetic analysis of the ensemble of *rap* genes (see Methods for details of the phylogenetic analysis and S3 and S4 Data). Although the overall divergence of Rap homologs was large, the family was clearly divided into two groups, corresponding to the division between the *B*. *cereus* and *B*. *subtilis* groups of species (Fig 2B). This suggests that the divergence of *rap* genes in each of these groups occurred after their evolutionary separation, with no horizontal transfer between groups. The phylogeny of the *B*. *clausii* and *B*. *halodurans* Rap proteins suggests that they acquired their *rap* genes by one or two horizontal gene transfer events, respectively, from *B*. *subtilis* group isolates, followed by intraspecific diversification and accumulation, as seen in the two major groups (Fig 2B). No evidence of divergence of the Rap protein through recombination of different Rap homologs was found (Methods). These observations suggest that diversification and paralogous expansion occurred independently multiple times during the evolution of the Rap-Phr system.

To gain further insight into the population genetics underlying the diversification of the Rap-Phr family, we focused on the *B*. *subtilis* group, in which multiple Rap-Phr systems have been previously characterized. The known Phr autoinducer sequences and the patterns of phr sequence conservation along the Rap phylogenetic tree were used to identify putative penta or hexa-peptide autoinducers and to cluster the Rap proteins (Fig 2C). All together, we defined 102 clusters with 81 unique Phr autoinducer peptides. To the best of our knowledge, this extreme autoinducer diversity is much greater than that observed in any other family of quorum-sensing systems.

In order to identify the mode of acquisition of a novel Rap-Phr system into a genome, we analyzed the level of horizontal gene transfer of Rap-Phr systems. We used two independent measures to estimate this trait—guanine-cytosine (GC)-content analysis and abundance analysis. First, because mobile element-related genes in *B*. *subtilis* typically have a significantly lower GC-content as compared to the rest of the genome [48], we characterized Rap-Phr as mobile if their GC-content was significantly lower than the average GC-content of their respective strain (Methods, S3 Fig). We found that 75% of Rap-Phr clusters were mobile (Fig 2C, bottom right). In parallel, mobile (or accessory) genes can be identified by their intermittent appearance within strains of a given species. Thus, we constructed an association matrix, in whcih each Rap cluster was marked as either present or absent in each of the genomes of *B*. *subtilis* group isolates (S4 Fig). With few exceptions, *rap*s identified as core genes by their GC-content appeared in the great majority of isolates from a given species, whereas mobile Rap systems appeared in only a few strains (S5 Fig, S5 Data), demonstrating a good correlation between the two measures of mobility. These results suggest that the acquisition of a novel Rap-Phr into a genome is dominated by horizontal gene transfer. To determine whether duplication, as the alternative mode of acquisition, occurred as well, we searched for cases in which two Rap-Phr systems from the same cluster were coded in the same genome (S4 Fig). Only five such cases were identified, three of which occurred in clusters that were categorized as mobile by both criteria above and may result from a recent duplicated introduction of a single mobile element. Therefore, there is little evidence for direct duplication events in Rap-Phr that belong to the “core” genome.

We noted that different species in the *B*. *subtilis* group have different core Rap paralogs (S4 Fig). The observed diversity pattern fits a slow ongoing process of fixation of Rap-Phr systems, in which some Rap variants (e.g., RapA and RapC) are fixed in multiple related species, while others are fixed only in a single species within the group. Interestingly, all orphan Rap systems belonged to the core group, by both modes of assessment, with RapD present in all but one species (*B*. *licheniformis*). Although mobile Rap-Phr systems dominated the population diversity, most Rap paralogs in any given strain (60% ± 10% mean ± st.dev.) belonged to the core group (Fig 2C, top right). Despite the abundance of available genomic data, we do not observe a saturation of Rap-Phr diversity with strain number (S6 Fig). The large diversity is also evident from the fact that several *rap-phr* clusters were found only on separately sequenced plasmids [49–52].

### Target Diversification: Rap Regulation of the ComA and Spo0A Pathways Has Been Gained and Lost Multiple Times

**Fig 3:**
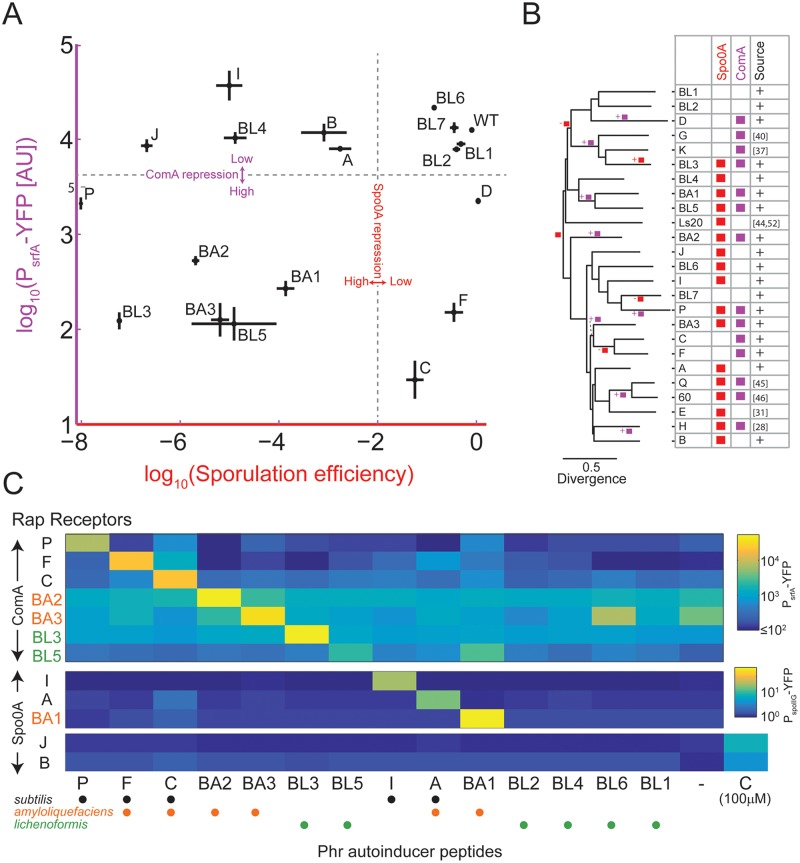
Target switching and autoinducer specificity of Rap-Phr pairs. (A) The effect of overexpression of multiple Rap systems on sporulation efficiency (*x*-axis) and on P_srfA_-YFP reporter expression (*y*-axis). Strains overexpressing *rapBA1-3*, *rapBL1-7*, and *rapI*, *P*, *B*, *J*, and *D* were constructed in a wild-type background, while strains overexpressing *rapF* and *rapC* were constructed in a *ΔrapFphrF; ΔrapCphrC* background. The effects of RapA were measured using a *ΔphrA* deletion mutant. To control for the indirect effect of the spo0A pathway on ComA activity, P_srfA_-YFP expression was measured in a background deleted for *spo0A*. See also S7 Fig. (B) Phylogenetic inference of Rap target evolution. Shown is the phylogenetic tree of all experimentally characterized Rap proteins. Shown on the right of the tree are the known effects of each of these Rap variants on Spo0A (red) or ComA (purple) activities. The source column is marked with a “+” sign if this interaction was identified or verified in this work, while a reference number is given otherwise. The GLOOME software [55] gain/loss parsimony results are marked on the tree. The root is marked by a red square to reflect the original Spo0A activity control. A branch marked with a square and “+” or “-”signs next to it indicates that the analysis established that a regulatory activity was gained or lost, correspondingly, along the specific branch. (C) Specificity of Rap-Phr systems. Yellow fluorescent protein (YFP) expression from a P_srfA_-YFP (top) or a P_spoIIG_-YFP (bottom) reporter in strains overexpressing the indicated *rap* genes (*y*-axis) and grown under appropriate conditions together with 10 μM of the indicated peptide phr autoinducer (*x*-axis, see S1 Table for peptide sequences. “-”indicates no peptide). For the orphans RapB and RapJ, *spoIIG* promoter activity was assayed also for the addition of 100 μM of phrC for comparison with previous works. Dots below each putative peptide indicate whether it is produced by isolates of the *B*. *subtilis* (black), *amyloliquefaciens* (orange), or *lichenoformis* (purple) species. Raw data for panels A and C are given in S5 Data.

Current data indicate that despite the large sequence and autoinducer diversity, many of the Rap receptors target either Spo0F, ComA, or both. The evolutionary rate of target specificity shift is unclear. To experimentally determine the specificity of multiple Rap systems, we used two isolates (marked in S4 Fig), *B*. *amyloliquefaciens* FZB42 and *B*. *licheniformis* ATCC 14580, as templates for the cloning of ten novel *rap* genes, most of them core genes of their respective species (Fig 3 and S4 Fig, S1 Table). These newly cloned Rap genes belong to previously unexplored branches of the Rap phylogeny. Correspondingly, these Rap homologs almost double the phylogenetic diversity [53] of characterized Rap proteins (from ~6% to ~10% of the total phylogenetic diversity of Rap proteins in the *B*. *subtilis* group). In addition, seven *B*. *subtilis*-associated *rap* genes (*rapC*, *F*, *I*, *P*, *J*, *B*, and *D*) were cloned under the control of the inducible hyper-spank promoter [54]. All genes were introduced into the genome of strain PY79. To prevent interference by the endogenous Phr product, the *rapC*, *F* genes were introduced into a strain with a deletion of these two systems (S7 Fig, S5 Data). Finally, an available *phrA* deletion was used to assay the effect of RapA.

The different Rap proteins were assayed for their effects on the Spo0A and ComA pathways by measuring their impact on sporulation efficiency and expression of the ComA-regulated *srfA* promoter using a yellow fluorescent protein (YFP) reporter [43], respectively (Fig 3A, S5 Data, Methods). Because ComA activity is indirectly affected by the Spo0A pathway [56], we introduced a *spo0A* deletion into each of the YFP-reporting strains.

Five out of the ten novel Rap proteins affected both sporulation efficiency and *srfA* expression, while RapBL4 affected only sporulation. Four of the novel rap overexpression constructs did not strongly affect either pathway. The eight *B*. *subtilis*-associated Rap proteins had the expected, previously characterized effect, with RapA, I, B, and J affecting Spo0A [26,33], RapF, C, and D affecting ComA [34,37], and RapP affecting both pathways [43].

These data allow us to better estimate the rate at which target choices change along the evolution of the Rap lineage. Based on our results and those reported by others, we assembled a phylogenetic tree of 25 Rap variants whose targets have been at least partially characterized (Fig 3B). We used the GLOOME program [55] to estimate the rate of gains or losses of regulation of the Spo0A and ComA pathways. We found the most parsimonious switching model to include 12 gain and loss events (Fig 3B), starting with an ancestral strain that regulated spo0A activity. This ancestral strain acquired the ability to control ComA in multiple independent events (see Discussion).

The interactions between Rap proteins with the aforementioned targets were recently analyzed at the structural level, allowing for the identification of specific Rap residues that directly interact with each target [27,28]. Upon analysis of the conservation of these residues in the characterized Rap proteins according to their functional targets (S8 Fig), we found that the amino acid residues, where RapF interacts with its target ComA, were not conserved in many other ComA-interacting Rap proteins. In contrast, the RapH amino acid residues at the interface with Spo0F were highly conserved in characterized Rap proteins, irrespective of whether they regulate the Spo0A pathway or not. The high level of conservation of Spo0F-interacting residues and low level of conservation of ComA-interacting residues were also demonstrated upon analysis of all *B*. *subtilis* group-associated Rap proteins using the ConSurf program (S9 Fig) [57]. These results further support the ancestral origin of the interaction between Rap proteins and Spo0F and the independent gain of ComA interaction by multiple sub-lineages of Rap proteins, as suggested by the parsimony analysis (Fig 3B).

### Autoinducer Diversification: Specificity of Interaction between Divergent Rap-Phr Systems

Anecdotal experiments in strain 168 have indicated that divergent Rap-Phr pairs are orthogonal—a receptor from one Rap-Phr strain will predominantly respond only to its cognate autoinducer [33,35,38,41]. This notion has not been studied systematically. It is also unclear whether divergent Rap-Phr systems encoded on different chromosomes would maintain orthogonality. We took advantage of the large collection of inducible Rap systems to thoroughly analyze these points. Fourteen custom-made putative autoinducer peptides were assayed for their ability to restore gene expression in the presence of different inducible Raps (Fig 3C). We used a peptide concentration of 10 μM, a level that exceeds both the measured affinity of Phr peptides to cognate Raps and the physiological levels of Phr (Methods) [25,30,43]. The interactions between peptides and Rap proteins were monitored using either the P_srfA_-YFP or P_spoIIG_-YFP reporter constructs, depending on whether the Rap targets the ComA or Spo0A pathways, respectively (Fig 3C, S5 Data). Rap proteins that affect both pathways were assayed only once.

We found that the repressive effect of all Rap proteins on gene expression was alleviated by addition of saturating amount of their respective cognate Phr peptide. One exception to this rule was RapBL4, which did not interact with its putative Phr pentapeptide (with amino-acid sequence GRAIF). We also found that the orphan RapB, J proteins were not affected by any Phr but that a 10-fold higher concentration of PhrC did activate them, in accordance with previous works that suggested this weak interaction (Fig 3C) [33,35].

We observed strict maintenance of orthogonality of Rap-Phr systems residing on the same genome. In two cases, cross-talk between two systems encoded by different strains was detected, with RapBL5 responding to both its cognate PhrBL5 autoinducer and to the related PhrBA1 autoinducer, and RapBA2 responding to its cognate PhrBA2 and more weakly to PhrBL6. Notably, the RapBL5 and RapBA1 proteins were only weakly divergent (Fig 3B), but RapBA2 and RapBL6 were unrelated. Our data therefore support the notion of strong orthogonality of Rap-Phr systems encoded in the same genome and some orthogonality between divergent Rap-Phr systems encoded by different genomes.

### Rap-Phr Divergence of Specificity through an Intermediate Promiscuous Duplicated Phr Form and Receptor Coevolution

**Fig 4:**
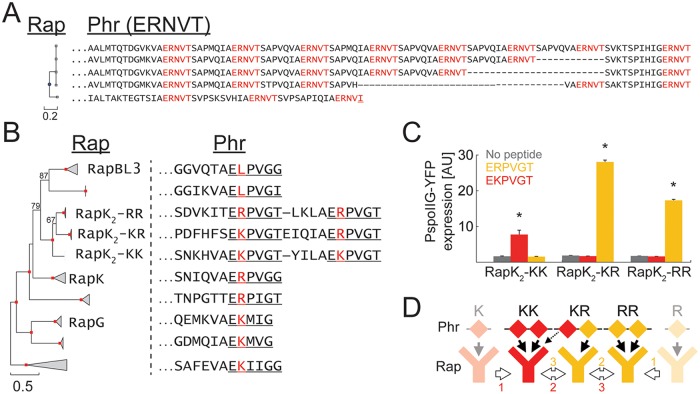
Phr sequence duplication and neofunctionalization. (A) Phylogenetic tree of five specific Rap variants (left) and their respective Phr sequence. The first twenty amino acids of the Phr sequence are omitted. Marked in red are repeated sequences of the putative Phr autoinducer. Underlined in the bottom sequence is a putative autoinducer sequence that deviates from the consensus autoinducer. (B) Phylogenetic tree of 140 Rap variants related to RapK. Triangles mark a cluster with the same putative autoinducer. Triangle lengths mark the mean distance between the ancestral node of the cluster to specific leaves. Red squares mark nodes with 100% support (out of 100 bootstrap repeats). Several additional nodes are marked by the number of bootstrap repeats supporting them. If a variant from a cluster has a name, it is marked next to that cluster. The C-terminal part of a representative cognate Phr sequence of each cluster is shown on the right. Underline marks the putative autoinducer peptide. The red letter marks the variable second residue of the putative autoinducer. (C) The YFP levels of a P_spoIIG_-YFP reporter integrated into strains over-expressing either RapK_2_-RR, RapK_2_-KR, or RapK_2_-KK. YFP was measured in the absence of peptide (gray) or following the addition of 10 μM of either ERPVGT (orange) or EKPVGT (red). See Methods and S5 Data for further details. Error bars mark standard errors. Asterisks mark cases in which peptide addition was significantly different from samples with no peptide. (D) Two possible evolutionary scenarios for the diversification of the RapK_2_ variants.

Thus far, our results suggest that the Phr peptides coevolve with their cognate Rap receptors to maintain the specificity of interaction, while Rap receptor affinity to its main two targets can change. To understand the evolution of the Phr peptides, we studied the diversity of the *phr* sequences in further detail. All Rap-Phr systems analyzed to date have a single *phr* gene, coding for a single Phr autoinducer penta- or hexa-peptide. In contrast, we found multiple cases in our database where peptide autoinducer coding regions were duplicated (Fig 4, S10 Fig). In some cases, all putative autoinducer repeats were identical (Fig 4A and S10E Fig), while in others, a single *phr* gene coded for multiple similar, but nonidentical, putative peptide autoinducers (Fig 4B and S10A–S10D Fig). For example, the Phr prepeptides of a group of closely homologous Rap proteins (related to RapH) contain multiple varying repeats of the motif [S/I][D/I/N/Y]RNT[T/I] (S10B Fig).

We also identified two subclusters of the *B*. *subtilis* Rap-Phr systems, in which the entire *phr* gene had undergone a duplication event (S10C and S10D Fig). The putative peptide autoinducers of the two *phr* genes had also diverged. We also observed sequence duplications (either intragenic or full-gene) events in *rap-phr* loci of other *Bacilli* (S11A–S11C Fig). A similar analysis of the related NprR-NprX quorum-sensing family [58,59] showed duplications in some *nprX* genes as well (S11D Fig, Methods). These results indicate that autoinducer duplications are abundant and that putative autoinducer sequences diverge after duplication.

Autoinducer duplication may facilitate the coevolution of Rap-Phr pairs by allowing a duplicated and diverged *phr* gene to serve as a transient promiscuous intermediate (Fig 1B). To experimentally examine this possibility, we analyzed a subset of Rap-Phr systems of the RapK/RapG/RapBL3 cluster (Fig 4B) [37]. The *phr* gene of three closely related systems in this cluster encodes for a pre-peptide with two putative autoinducer peptides. One system, designated RapK_2_-RR, encodes twice for the putative penta- or hexapeptide ERPVG(T). The second system (RapK_2_-KR) encodes for the putative autoinducers ERPVG(T) and EKPVG(T), while the third (RapK_2_-KK) encodes twice for the putative autoinducer EKPVG(T). RapK_2_ variants (S1 Table) were cloned under the control of the hyper-spank promoter and monitored for the effect of their overexpression on a P_spoIIG_-YFP reporter, as described above (Fig 4C, S5 Data). *spoIIG* promoter activity was repressed to background levels in all three overexpression strains. *spoIIG* promoter activity was restored in strains overexpressing either RapK_2_-RR or RapK_2_-KK upon addition of their cognate hexapeptides (ERPVGT and EKPVGT, respectively), but not when their non-cognate hexapeptide was added (Fig 4C). RapK_2_-KR, whose cognate Phr encodes both type of peptides, responded only to the addition of ERPVGT. None of the strains responded to the addition of the relevant pentapeptides. The specificity shift between the different RapK_2_ variants may therefore provide an example for the role of duplication in such an event.

## Discussion

### A Separate Role for Horizontal Gene Transfer and Duplication during the Expansion of the Rap-Phr Family

**Fig 5:**
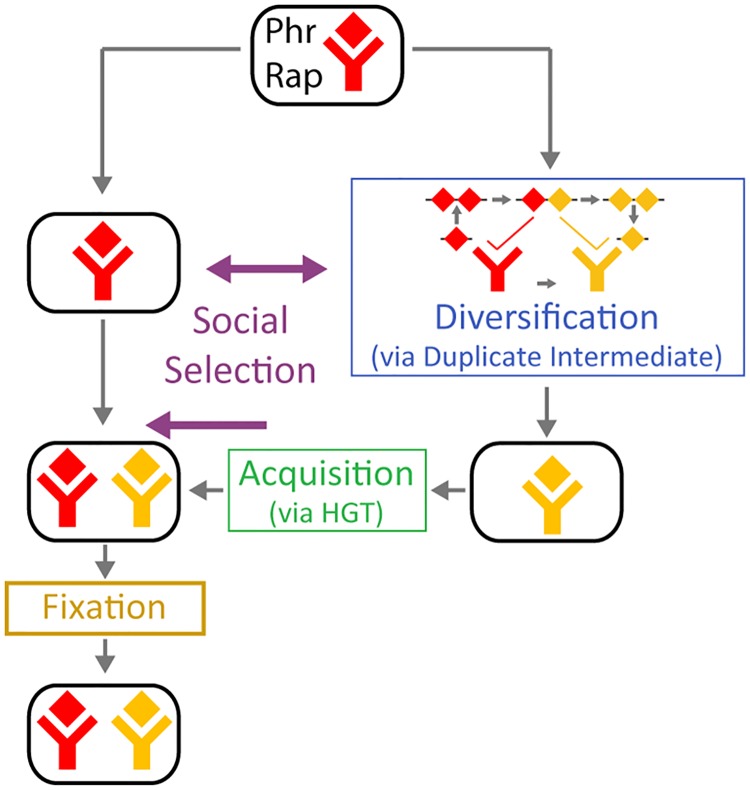
Evolutionary dynamics of the Rap-Phr pathway. Rap-Phr expansion combines a horizontal mode of acquisition—systems diverge in separate lineages and then recombine by HGT—with a vertical mode of divergence—an autoinducer is duplicated and then one of the duplicates is modified. Social interactions govern both diversifying selection between lineages and acquisition of a diverged system [41,60,61]. Acquisition by horizontal transfer is sometimes followed by fixation in the lineage.

The diversity and functional orthogonality of diverging signal transduction paralogs in bacteria are well characterized [9,11,12]. However, the way by which new paralogs are acquired and diversified and, specifically, the roles of horizontal and vertical events in this process are not clear. In this work, we showed that both horizontal and vertical processes are crucial for the expansion of the Rap-Phr quorum-sensing system but operate at different levels of organization. Acquisition of a novel Rap-Phr system was shown to be facilitated by horizontal gene transfer, while diversification was facilitated by *phr* duplications within the diversifying locus (Fig 5).

A key novelty of our finding is exposure of the role of duplications in the divergence of Rap-Phr system specificity. The prevalence of intragenic and whole-gene *phr* duplications and the experimental analysis of the RapK_2_-PhrK_2_ variants (Fig 4 and S10 and S11 Figs) suggest that transient Phr duplication and divergence play a role during evolutionary shifts in Rap-Phr specificity (Fig 4D). The ancestral copy of Phr interacts with the ancestral Rap form, while the duplicated and diverged autoinducer copy has the potential to interact with a coevolved receptor. Importantly, this mechanism differs in two aspects from the common views of pathway diversification. First, divergence of a signaling pathway is typically linked with a promiscuous protein that can interact with the two forms of its partner [2,12]. Here, the promiscuous form is the duplicated *phr* and not a single Phr peptide, which interacts with both Rap variants. Second, duplications are typically only considered important if both diverged duplicates survive over evolutionary timescales [3]. In contrast, Phr duplication and divergence are evolutionarily crucial for a specificity shift, but to complete the shift it has to be transient—with either divergence or duplication itself being lost. In the specific case we examined, the duplication persisted, while duplicate diversity was transient (Fig 4D).

### Rapid Divergence of Rap Interaction with Phr and Downstream Targets

We found that Rap acquisition and divergence occurred independently in multiple evolutionary lineages, indicating that it is an intrinsic feature of the function of this system. In the *B*. *subtilis* group alone, we identified dozens of putative Phr autoinducer peptides, rendering it the largest known quorum-sensing family. While the high orthogonality of a significant number of pairs was experimentally verified (Fig 3B), further experimental work will be required to explore the level of orthogonality between all clusters. In fact, some cases of cross-interactions were detected (Fig 3C). Notably, strong cross-interactions between Rap-Phr pairs encoded in the same genome were not observed, while weak interactions, as seen between PhrC and the orphans RapB and J (Fig 3C) [33,35], or RapF [41], were noted. In general, the functional importance of nonspecific interactions is unclear. More specifically, the interaction between orphan Raps and non-cognate Phrs may be physiologically irrelevant, given the low affinity of these interactions (~100 μM) compared with the physiological concentration of Phrs (~100 nM) [25]. Whether orphan Raps interact with other signaling molecules remains to be determined. Notably, formation of orphan Raps is rare, and all major *B*. *subtilis* orphan Raps are anciently fixed in their genome (Fig 2C and S4 Fig).

Rap proteins also diverge with regards to the targets they regulate. Our data indicate that the distinction between targets is not the result of ancient diversification but rather is the result of an ongoing process of multiple events of gain and loss of target regulation (Fig 3A and 3B). The GLOOME parsimony analysis indicated that the ancestral Rap receptor regulated the Spo0A pathway and that the regulation of ComA by Rap proteins has been gained multiple times within the *B*. *subtilis* group. This is in agreement with the low sequence conservation between different ComA-regulating Rap variants (S7 and S8 Figs) and the absence of ComA homologs in the majority of *B*. *cereus* strains. In addition, the mechanism of ComA regulation differs across Raps. RapF blocks comA binding to DNA through direct competition for the DNA binding domain [27]. In contrast, Rap60 does not block ComA DNA binding but prevents the DNA-bound ComA from activating transcription [46].

Phr diversification is accompanied by coevolution of the Rap receptor. A key future challenge is to identify the amino-acid positions of Rap that are crucial for its coevolution and the underlying structural principles of peptide-receptor specificity. Our results suggest that even key conserved features of this interaction can be lost during diversification. Specifically, arginine at the second position of the Phr autoinducer is highly conserved due to a salt-bridge with a conserved negatively charged residue on the Rap protein (Fig 2) [32,33]. While substitution to lysine (as in PhrG or PhrK_2_-KK) does not dramatically interfere with this interaction, this residue is substituted with the uncharged leucine in PhrBL3. Correspondingly, the conserved Rap aspartate residue is substituted by the non-charged glutamine residue in RapBL3, suggesting that the electrostatic interaction has been replaced by another type.

### Rap-Phr and the Diversification of Kind-Discrimination Systems

The mobile nature of the majority of Rap-Phr systems (Fig 2C and S3 and S4 Figs) and their functional adaptive role in the transfer of mobile elements [47,52] and in social interactions [41] indicate that they act as “kind-discrimination” systems [62]. Such systems mediate discriminative interactions between bacteria or between their genetic parasites. Kind-discrimination can operate through various mechanisms such as cell–cell signaling [60], intracellular toxin-antitoxin [63], aggregation [64,65], surface exclusion [66], bacteriocin-immunity [67], or contact-mediated toxins [68,69]. Most kind-discrimination systems are two-gene systems with a high divergence of specificity between interacting pairs. Like the Rap-Phr system, some of these systems tend to accumulate in large numbers within bacteria [70]. Notably, the social nature of kind-discrimination implies that alleles may strongly interact even if they are not encoded in the same bacterium. This presumably increases selection pressure for specificity (Fig 5).

Intralocus duplication events can yield a transient promiscuous intermediate in other kind-discrimination systems. A recent analysis showed that toxin-antitoxin systems can shift specificity through promiscuous toxin intermediates, which can mediate interactions with two different antitoxins [12]. However, this does not rule out the possibility of duplication as an alternative mechanism. Further analysis of natural variation will be required to further assess these phenomena. One difference between Rap-Phr and other kind-discrimination systems is the short length of both the phr *gene* and the mature autoinducer peptide. This may promote duplication and neofunctionalization in peptide-based quorum-sensing systems in comparison to other systems where both interacting partners are larger globular proteins. Interestingly, fungi mating pheromones show a striking similarity to the Phr diversity, with both genic and intragenic duplications of the pheromone peptide coding sequences as well as some cases where there is sequence variability between duplicates [71]. Duplications may arise by sexual selection [72], and it has been suggested, but not proven, that they may facilitate diversification.

Taken together, the data from this work and previous works [41,47] show how duplication and rapid horizontal transfer can work together to rapidly expand a signaling pathway family operating at multiple levels of selection. Further work will be needed to determine the generality of this phenomenon.

## Methods

### Experimental Methods

#### Growth media, conditions, and reagents

Routine growth was performed in Luria–Bertani (LB) broth: 1% tryptone (Difco), 0.5% yeast extract (Difco), 0.5% NaCl. Experiments in which P_srfA_-YFP expression was measured were done using Spizizen minimal medium (SMM): 2 g L^−1^ (NH_4_)_2_SO_4_, 14 g L^−1^K_2_HPO_4_, 6 g L^−1^KH_2_PO_4_, 1 g L^−1^disodium citrate, 0.2 g L^−1^MgSO_4_·7H_2_O. This was supplemented with trace elements (125 mg L^−1^MgCl_2_·6H_2_O, 5.5 mg L^−1^CaCl_2_, 13.5 mg L^−1^FeCl_2_·6H_2_O, 1 mg L^−1^MnCl_2_·4H_2_O, 1.7 mg L^−1^ZnCl_2_, 0.43 mg L^−1^CuCl_2_·4H_2_O, 0.6 mg L^−1^CoCl_2_·6H_2_O, 0.6 mg L^−1^Na_2_MoO_4_·2H_2_O). Glucose at 0.5% weight to volume ratio was used as carbon source. Experiments in which sporulation efficiency or P_spoIIG_-YFP gene expression was measured were done using Schaeffer's sporulation medium (DSM) [73]. Petri dishes for routine procedures were solidified using 1.5% agar (Difco).

Antibiotic concentrations: Macrolides-lincosamides-streptogramin B (MLS; 1 μg ml^−1^ erythromycin, 25 μg ml^−1^ lincomycin); Spectinomycin (Sp, 100 μg ml^−1^); Tetracycline (Tet, 10 μg ml^−1^); Kanamycin (Km, 5 μg ml^−1^); Chloramphenicol (Cm, 15 μg ml^−1^); Ampicillin (Amp, 100 μg ml^−1^). Isopropyl β-D-thiogalactopyranoside (IPTG, Sigma) was added to the medium at the indicated concentration when appropriate.

Pre-measurement *Bacillus* growth protocol: Prior to all measurements, an overnight colony from an LB agar plate was inoculated in 1 mL SMM liquid medium and grown for 7 h until an OD_600_ of 0.1–0.3 was reached. The cultures were diluted by a factor of 10^6^ and grown overnight at 37°C. Overnight cultures were centrifuged, resuspended in PBS, and diluted to an OD_600_ of 0.01.

Synthetic peptides were purchased from GL Biochem (Shanghai, China) at >98% purity. Peptide aliquots at a concentration of 10 mM were prepared by resuspension of the lyophilized peptides in H_2_O and stored at -20°C. Peptides used are listed in S1 Table.

#### Gene expression analysis

The cells were grown in SMM to an OD_600_ of 0.1, diluted by a factor of 10^6^ in fresh SMM, and grown overnight for about 20 h. For *P*_*srfA*_*-yfp* analysis, the samples were diluted to an OD_600_ of 0.1, grown for 2.5 h, and the appropriate peptides were added. After 1.5 h of incubation, the OD600 and YFP level were measured using a Perkin Elmer 2030 multilabel reader Victor x3. The results are presented as the YFP divided by the optical density. For P_spoIIG_-yfp analysis the overnight cultures were diluted to an OD_600_ of 0.01 in DSM medium. The appropriate peptides were added in two sequential steps of 1.5 h (at equal concentrations), after which flow cytometry was used to quantify gene expression at the single-cell level using a Beckman-Coulter Gallios system with a 488-nm laser. A minimum of 20,000 cells were analyzed for each sample. The results are presented as the mean YFP level of the population.

#### Sporulation efficiency assay

The cells were grown in SMM to an OD_600_ of 0.1, diluted by a factor of 10^6^ in fresh SMM, and grown overnight for about 20 h. The cultures were diluted to an OD_600_ of 0.01 in DSM medium and grown for 24 h. Serial dilutions were performed and samples were plated before and after heating (20 min at 80°C). Sporulation efficiency was calculated as the CFU after heating divided by CFU before heating.

#### Strain construction

All of the mutations and constructs were transferred to PY79 by transformation [73]. Integration of *amyE* integration plasmids into the *zjd89*::*amyEΩCmKm* [74] was done as previously described [43]. All strains used in this work are listed in S2 Table, and the corresponding primers used are listed in S3 Table.

Deletion of *rapK-phrK*, *rapF-phrF*, *rapC-phrC*, *phrA*, and *comA* from the PY79 chromosome and their replacement with an antibiotic Erythromycin resistance cassette was performed through the long flanking homology PCR method [75] using the primers rapK-P1-P4 rapF-P1-P4, rapC-P1-P4, phrA-P1-P4, and comA-P1-P4, respectively (S2 Table). The *rapFphrF*::Cm deletion was generated using the antibiotic switching vector ece76. *rapFphrF*::Cm was next used as a template to generate *rapFphrF*::Tet using the antibiotic switching vector ece75 (S2 Table).

To generate inducible *amyE*::*(P*_*hyperspank*_*-RAP)* constructs, a PCR product containing the relevant open reading frame (S1 Table) was amplified using the appropriate primer pair (S3 Table). The PCR products were digested with the appropriate enzymes (S3 Table) and ligated downstream of the hyper-spank promoter of the pDR111 vector containing Spec resistance [76].

Construction of *sacA*::(P_*srfA*_-3x*yfp* Cm) was performed by PCR amplification of P_*srf*_-3xyfp using AEC945 as a template and the Psrf-sacA-F/Psrf-sacA-R primer pair. The PCR fragment was digested with the appropriate enzymes (S3 Table) and ligated to the ece174 plasmid. The resulting vector was integrated into the *sacA* site on the chromosome using Cm resistance for selection.

Construction of *sacA*::(P_*spoIIG*_-3x*yfp* Cm) was performed by PCR amplification of the *spoIIG* promoter region using the PspoIIG-F/PspoIIG-R primer pair. The PCR fragment was digested with the appropriate enzymes (S3 Table) and ligated to the ece174 P_*srfA*_-3x*yfp* plasmid, replacing the *srfA* promoter region with the *spoIIG* promoter region.

### Computational Methods

#### Construction of Rap database and phylogenetic tree

All computational procedures were done using the Matlab bioinformatics toolbox (Mathworks) unless otherwise written. We downloaded 413 genomes from the *Bacillus* genus available at the time in the bacterial draft database of the NCBI. We used a local BLAST program to perform a BlastN search of each genome against four different Rap proteins: three Rap proteins from *B*. *subtilis—*RapA, RapC, and RapD—and one from *B*. *cereus* (accession numbers NP_389125, NP_388259, NP_391519, and NP_846019 respectively) [39]. An expectation threshold smaller than 1e-20 for any of the four Rap proteins was used to determine whether the BLAST results correspond to a true Rap protein. Importantly, this did not mix Rap proteins with their closest related family: the NprR family. In addition, we restricted the search only to cases in which the length of homology was larger than 300 amino acids. Cases of high homology with shorter length were browsed manually to determine whether this is due to a frameshift mutation in the Rap protein. Following the identification of Rap protein candidates, we searched for a full open reading frame associated with each Rap protein. This method led to identification of ~2,700 Rap homologs in >300 genomes, as described in the text.

Calculation of Rap phylogenetic tree was done as follows. All Rap proteins with full open reading frames were multiply aligned using mafft [77], with default parameters. The phylogenetic tree was built using RAxML, using the PROTGAMMA model with the LG substitution matrix [78]. One hundred fast bootstrap replicates were calculated, and 20 of the resulting trees were used as seeds for a more thorough ML optimization. When specifically mentioned, phylogenetic tree was also calculated with the PHYML, maximum-likelihood based program [79].

Recombination analysis of Rap proteins was performed using the RDP3 program, which was used with default parameters. We searched for clear cases in which a Rap protein in the database is identified as a recombination product of two other Rap proteins existing in the database.

Construction of isolate evolutionary phylogeny was done using Matlab by BLAST identification, multiple alignment, and tree construction based on the GyrA protein sequence, with similar parameters to those described above. This phylogeny led to the same phylogenetic tree identified by others [80] and was sufficiently good for our analysis. We therefore did not refine the phylogeny further by concatenating additional genes.

#### Identification of putative phr genes

For each Rap protein, we searched for short (35–120aa) open reading frames in the DNA sequence, which starts 100 bp prior to the end of the Rap open reading frame and ends 600 bp after it. All identified reading frames were scored for the existence of a secretion signal sequence using the PrediSi program [81]. By comparing the quality score of the sequence of known *phr* genes, we set a threshold of 0.3 for positive identification of a secretion signal sequence. Candidate *phr* genes were further screened manually based on their conservation pattern. Putative Phr autoinducer peptide sequences were identified manually based on patterns of conservation and similarity to known Phr autoinducer peptides. The putative Phr signals were used to construct the clustered phylogenetic tree shown in Fig 2C.

#### Gain/loss analysis of the regulation of ComA and Spo0A pathways

We binarized the data presented in Fig 3A based on the thresholds presented as dotted lines in the figure to determine whether each Rap represses the Spo0A and ComA pathways or not. We used this binary representation and the phylogenetic tree of the indicated Rap proteins as an input for the GLOOME program [55], assuming equal likelihood of gains and losses and other default parameters. We used the maximum parsimony option to calculate the most likely state of each internal node of the phylogenetic tree.

#### Conservation analysis of ComA- and Spo0F-associated residues

We used the multiple alignment of *B*. *subtilis* group analyzed Rap proteins to identify the sequence motifs shown in S8 Fig. In addition, the ConSurf program [57] was used with default parameters to analyze the conservation of residues using the multiple alignment of all Rap proteins of the *B*. *subtilis* group and display them on the structure of the RapF protein in complex with ComA DNA binding domain (PDB ID 3ulq) and of RapH in complex with Spo0F (PDB ID 3q15) (S9 Fig).

#### Analysis of NprR quorum-sensing system

This analysis was done similarly to that performed for Rap proteins. An NprR amino-acid sequence (S1 Table) was used to perform a BlastN search on all genomes. We only considered genomes with a full homolog of NprR with an expectation number smaller than 1e-30. To identify putative *nprX* genes, we searched for a short open reading frame upstream of the *nprR* homolog open reading frame (from -100 bp to +500 bp of the end of the *nprR* open reading frame). Sequences were checked for their secretion signal sequence motif and compared with published NprX sequences [59]. Phylogenetic tree was constructed in the same manner described above for the Rap proteins.

## Supporting Information

S1 Fig Phylogeny of Rap encoding *Bacilii*. A phylogenetic tree (based on the GyrA gene) of different species in the Bacillus genus. Indicated in red are species where Rap homologs have been identified. The number in parenthesis indicates the number of isolates of any given species. The *Bacillus subtilis* group of species is indicated.
(TIF)

S2 Fig Genetic organization and function of the Rap-Phr locus. (A) A scheme of the organization of a typical *rap-phr* locus. The *rap* gene is followed by a *phr* gene, which is driven both the bicistronic *rap* promoter and sometimes by an internal promoter located within the *rap* gene. Shown are the coding regions for the 3-helix bundle and TPR domain of the Rap protein and the coding region for the secretion signal sequence and for the autoinducer. Notably, in some annotated *phr* genes (*phrH*, *phrE*), the autoinducer sequence is not at the C-terminal of the Phr prepetide, but is followed by additional amino-acids. (B) A scheme of the Rap-Phr system function. Phr prepeptide is produced,cleaved during secretion,and then cleaved once or more extracellularly. The mature autoinducer peptide is imported, through the Opp system, back to the cytoplasm, where it interacts with a cognate Rap receptor. Rap receptors repress ComA or Spo0F activity (and in some cases other targets). This repression is prevented upon phr autoinducer binding. The *rap-phr* operon is often controlled by ComA, while the internal *phr* prmoter is controlled by Spo0A.
(TIF)

S3 Fig Variation in *rap* %GC content correlates with the *rap* neighborhood %GC. For each *rap* gene of the *B*. *subtilis* group, %GC content and the %GC content of the DNA sequence encoded from 1kbp to 300bp upstream to the *rap* start codon were calculated. A clear correlation was observed between the two %GC content measures (R^2^ = 0.69). Marked is the best-fit line (y = 1.1x).
(TIF)

S4 Fig Shown are the phylogenetic trees of Rap clusters (left, a linear representation of the tree presented in Fig 2C, red lines mark cluster mean distance, as triangles do in Fig 2C) and the *Bacillus subtilis* group strains(top, based on the GyrA sequence). The presence of a Rap protein of a certain cluster in a given strain is marked by a colored rectangle, where the colors indicate whether the cluster is considered horizontally transferred (yellow) or fixed (red), based on the %GC content of the *rap* sequence (corresponding to background color of the cluster in Fig 2C). A diagonal line over the specific rectangle marks cases where two duplicates from the same cluster are found in a single genome (3 cases for orphan RapJ and 5 cases for Rap-Phr systems). Species are separated by thicker vertical lines and species names are marked below the matrix. The three strains which were used for experiments are specifically marked. Putative Phr autoinducers that define each cluster are marked to the right of the matrix. The Rap name is given as well. Names of newly characterized Rap proteins are given in cyan. Matrix column and row numbers are shown in intervals of 10. The relation between column number and strain info and between row number and cluster info are presented in S1 Data, tabs 3,4.
(TIF)

S5 Fig Relation between Rap-Phr locus frequency and %GC content. For each cluster present in the major species (*B*. *subtilis*, *B*. *amyloliquefaciens*, *B*. *licheniformis* and *B*. *pumilus*), the frequency of strains of each species where a *rap* gene belonging to this cluster exists was calculated (S5 Data). If a representative of a given cluster existed in more than one of the species, their frequency in the different species was averaged. Shown are the histograms of frequencies for clusters which were characterized as core (red) or as mobile (yellow), based on their %GC content. Clearly, mobile clusters mostly occur at low frequencies, while core clusters mostly occur at high frequencies.
(TIF)

S6 Fig Rap-Phr cluster diversity is not saturated despite the large genome number. Shown are the average number of clusters identified as a function of the size of the group of strains examined. For each group size, 500 random samples were examined. The curve does not saturate even at maximal strain number. For the last 20 strains, the slope of newly identified clusters is ~0.2 clusters per new strain.
(TIF)

S7 Fig The effects of Rap overexpression. (A) Sporulation and (B) P_srf_-YFP expression following expression of different Rap constructs (repeat of the data shown in Fig 3A of the main manuscript, S5 Data). Two additional controls, not shown in Fig 3A, are the sporulation efficiency of strains *ΔcomA* and *ΔrapFphrF;ΔrapCphrC*. A single asterisk indicates p<0.05, while two asterisks mark p<0.001 (two sample t-test between the strain and its parental background which lacks the Rap overexpression construct).
(PDF)

S8 Fig Sequence conservation of Spo0F- and ComA-interacting residues. Sequence alignment of experimentally characterized Rap proteins at residues which were inferred to interact with Spo0F and ComA, based on the crystal structure of RapH in complex with Spo0F (green) [1] and of RapF in complex with the ComA DNA binding domain (red) [2]. Residue numbers and consensus are shown above the alignment. For each target, we calculated the consensus for sequences that affect the specific target (designated Target) and for sequences that do not affect it (designated OFF-Target).
(TIF)

S9 Fig CONSURF conservation analysis for ComA- and Spo0F-interacting residues. Output results of a Consurf analysis performed on the alignment of all *B*. *subtilis* group Rap proteins using the structure of (A) RapF in complex with the ComA DNA binding domain (PDB ID: 3ulq), or (B) RapH in complex with Spo0F (PDB ID: 3q15). ComA and Spo0F are shown in a stick representation at the left side (A), or the middle (B) of the respective Rap proteins. The Rap proteins are depicted using a space-filling view, where each residue is marked according to its level of conservation based on the legend shown in (A). Stick representation at the right of RapH in (B), corresponds to a RapH dimer appearing in the crystal structure.
(TIF)

S10 Fig Additional examples of duplications of putative autoinducer sequences in the *B*. *subtilis* group. Shown are five more subtrees of the B. subtilis group Rap phylogeny, here some of the putative phr sequences encode multiple repeats of the autoinducer, with or without divergent amino acids. The subtree is based on the Rap amino acid sequence as presented in Fig 2C. Each leaf is marked with the sequence of the Phr prepeptide. Three dots indicate that the initial 20–30 amino acids (constituting the secretion signal sequence) are deleted. Putative autoinducer-like sequence are underlined. In (C,D) some of the *rap* genes are associated with two phr genes. In these cases, both sequences are shown. The scale bar marks the degree of amino acid divergence.
(TIF)

S11 Fig Autoinducer duplications in Rap-Phr outside of the B. subtilis group and the NprR-NprX system. (A) Phylogenetic relation between the six Rap proteins encoded in the single fully sequenced *B*. *clausii* isolate. All of these Rap proteins are monophyletic within the *B*. *subtilis* Rap sub-tree (Fig 2B). The putative autoinducer sequence is underlined. (B,C) Two examples of *rap* sub-trees from *B*. *cereus*. The putative Phr autoinducer is underlined. Duplicated parts of the pre-peptide are shown with curly brackets. Putative autoinducer sequence identification was based on ref. [3]. Sequences are aligned according to the best match between duplicates in different species. Note the small divergence scale of the two systems. Putative Phr sequences are unknown for *B*. *cereus* and are therefore not marked. (D) Phylogenetic tree of NprR variants from *B*. *cereus* group and other species. If the species from which the Rap sequence was taken is not part of the *B*. *cereus* group *sensu stricto*, its name is marked in parentheses. NprX prepeptide sequence is shown and the putative autoinducer sequence is marked [4,5]. Three dots mark the absence of the secretion signal sequence from the pre-peptide sequence.
(TIF)

S1 Table Data on newly cloned Rap systems and their respective peptides. (DOCX)

S2 Table Strain list. (DOCX)

S3 Table Primer list. (DOCX)

S1 Data Rap Database and other information. The Excel file contains five tabs. **(Tab 1)** Excel representation of the Rap Database, which is also provided as a Matlab structure file (S2 Data). Shown are index number, associated strain number, Clade, associated cluster number, nucleotide sequence, amino-acid sequence, Putative Phr autoinducer sequence (for B. subtilis group clade), Phr amino-acid and DNA sequence. Note that the Matlab file contain few additional data fields (see Tab 5). **(Tab 2)** Strain list with strain index number (used in the Rap database) and NCBI RefSeq number of the first sequence file. **(Tab 3)** The position of strains in the phylogeny presented in S4 Fig (top). **(Tab 4)** The position of cluster numbers in the cluster phylogeny shown on S4 Fig (left). **(Tab 5)** Identification and annotation of the fields of the Matlab Rap Database.
(XLS)

S2 Data Rap database file (Matlab). Matlab file containing the structure variable RapDataBase. The fields of the structure are explained in S1 Data.
(MAT)

S3 Data Rap (and NprR) phylogeny in Newick form. Phylogeny of 2896 amino-acid sequences used in Fig 2 of the main manuscript. Each leaf is designated by the Rap database number of the specific leaf. Each node is designated by a number followed by the support of the node in 100 bootstrap runs.
(TXT)

S4 Data Rap multiple alignment. Multiple alignment of Rap sequences used to produce the phylogeny of S3 Data (shown in Fig 2). Fasta format. Header marks database number of the Rap sequence.
(FASTA)

S5 Data Experimental data required for construction of Figs 2A, 3A and 3C, 4C, S5 and S7. (XLSX)

## Abbreviations

GC: guanine-cytosine
NCBI: National Center for Biotechnology Information
st.dev.: standard deviation
YFP: yellow fluorescent protein
