# Supplementary figures and images for: Transient Duplication-Dependent Divergence and Horizontal Transfer Underlie the Evolutionary Dynamics of Bacterial Cell–Cell Signaling
Source: PLoS Biol. 2016 Dec 29;14(12):e2000330. doi: 10.1371/journal.pbio.2000330 (PMC5199041; doi:10.1371/journal.pbio.2000330)

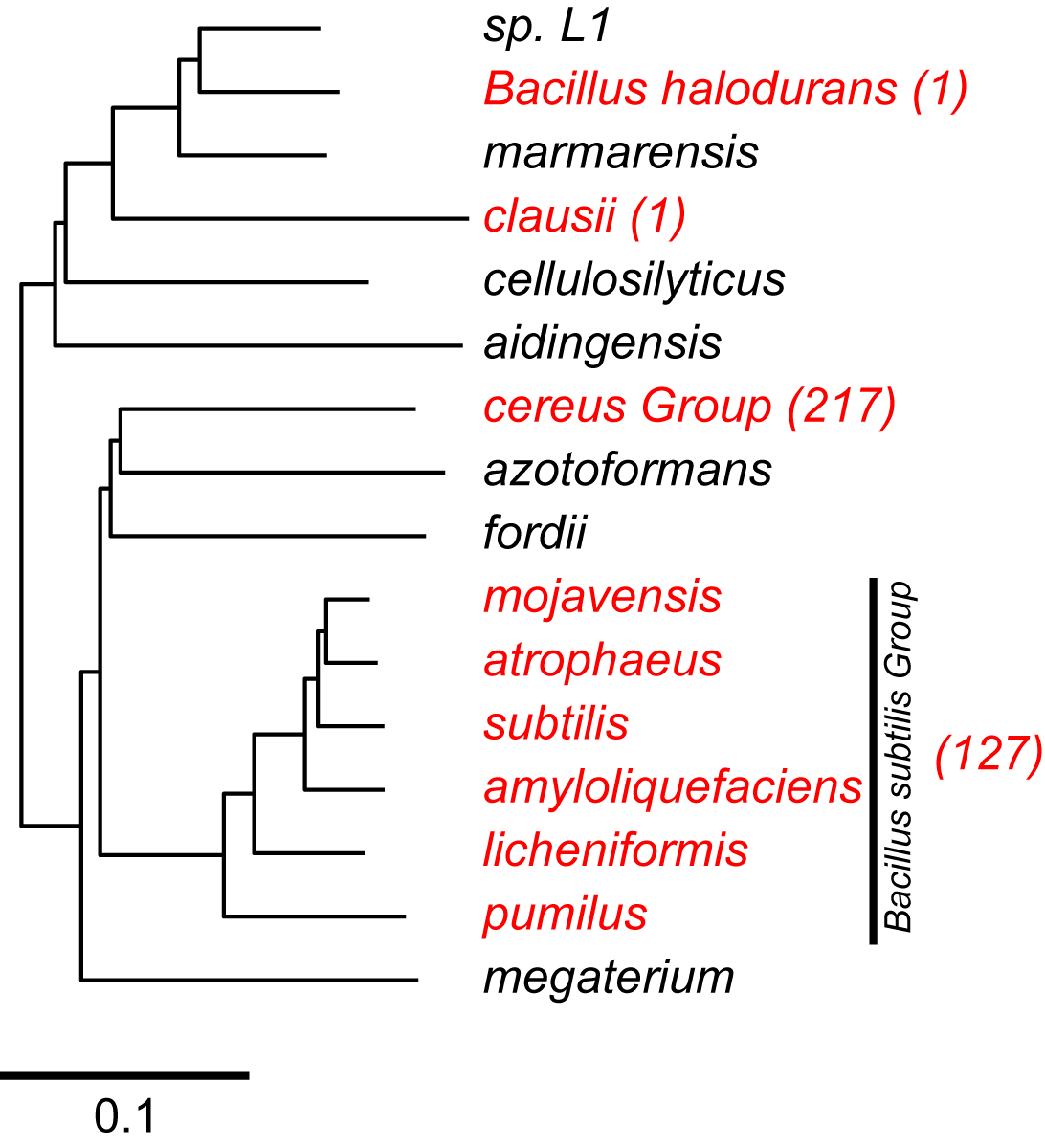

Supplement: S1 Fig — A phylogenetic tree (based on the GyrA gene) of different species in the Bacillus genus. Indicated in red are species where Rap homologs have been identified. The number in parenthesis indicates the number of isolates of any given species. The Bacillus subtilis group of species is indicated. (TIF) [file pbio.2000330.s001.tif]

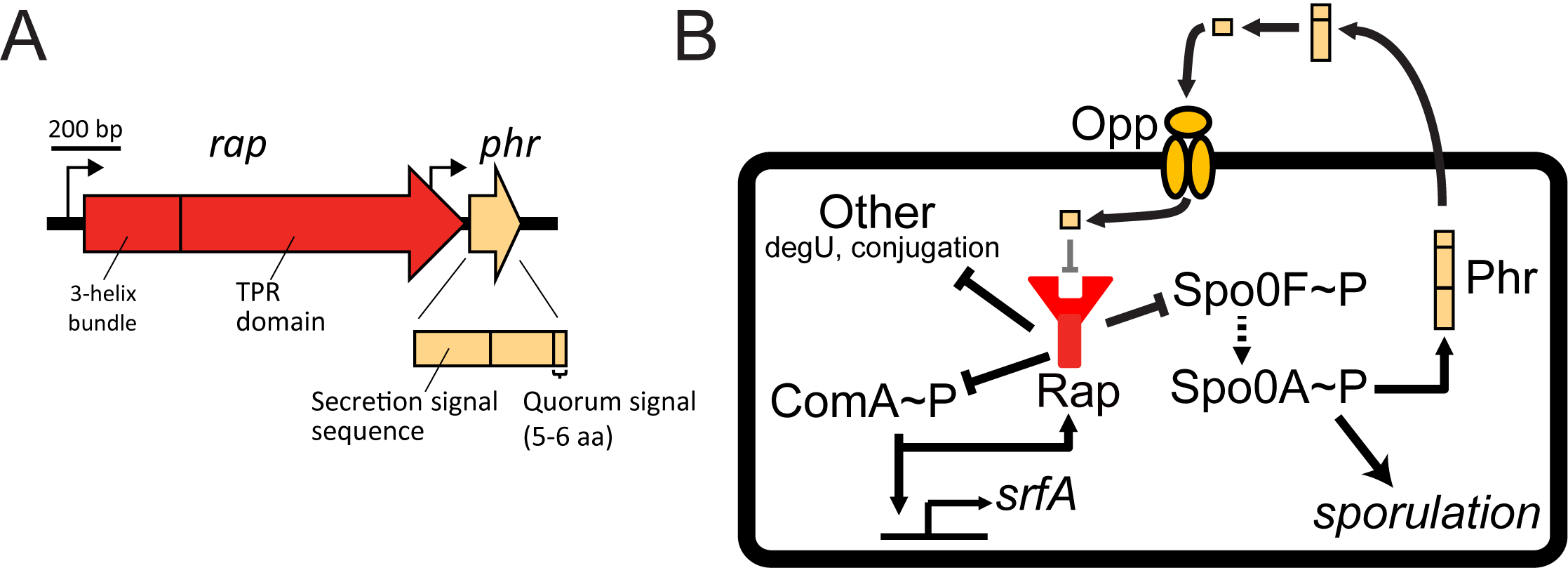

Supplement: S2 Fig — (A) A scheme of the organization of a typical rap-phr locus. The rap gene is followed by a phr gene, which is driven both the bicistronic rap promoter and sometimes by an internal promoter located within the rap gene. Shown are the coding regions for the 3-helix bundle and TPR domain of the Rap protein and the coding region for the secretion signal sequence and for the autoinducer. Notably, in some annotated phr genes (phrH, phrE), the autoinducer sequence is not at the C-terminal of the Phr prepetide, but is followed by additional amino-acids. (B) A scheme of the Rap-Phr system function. Phr prepeptide is produced,cleaved during secretion,and then cleaved once or more extracellularly. The mature autoinducer peptide is imported, through the Opp system, back to the cytoplasm, where it interacts with a cognate Rap receptor. Rap receptors repress ComA or Spo0F activity (and in some cases other targets). This repression is prevented upon phr autoinducer binding. The rap-phr operon is often controlled by ComA, while the internal phr prmoter is controlled by Spo0A. (TIF) [file pbio.2000330.s002.tif]

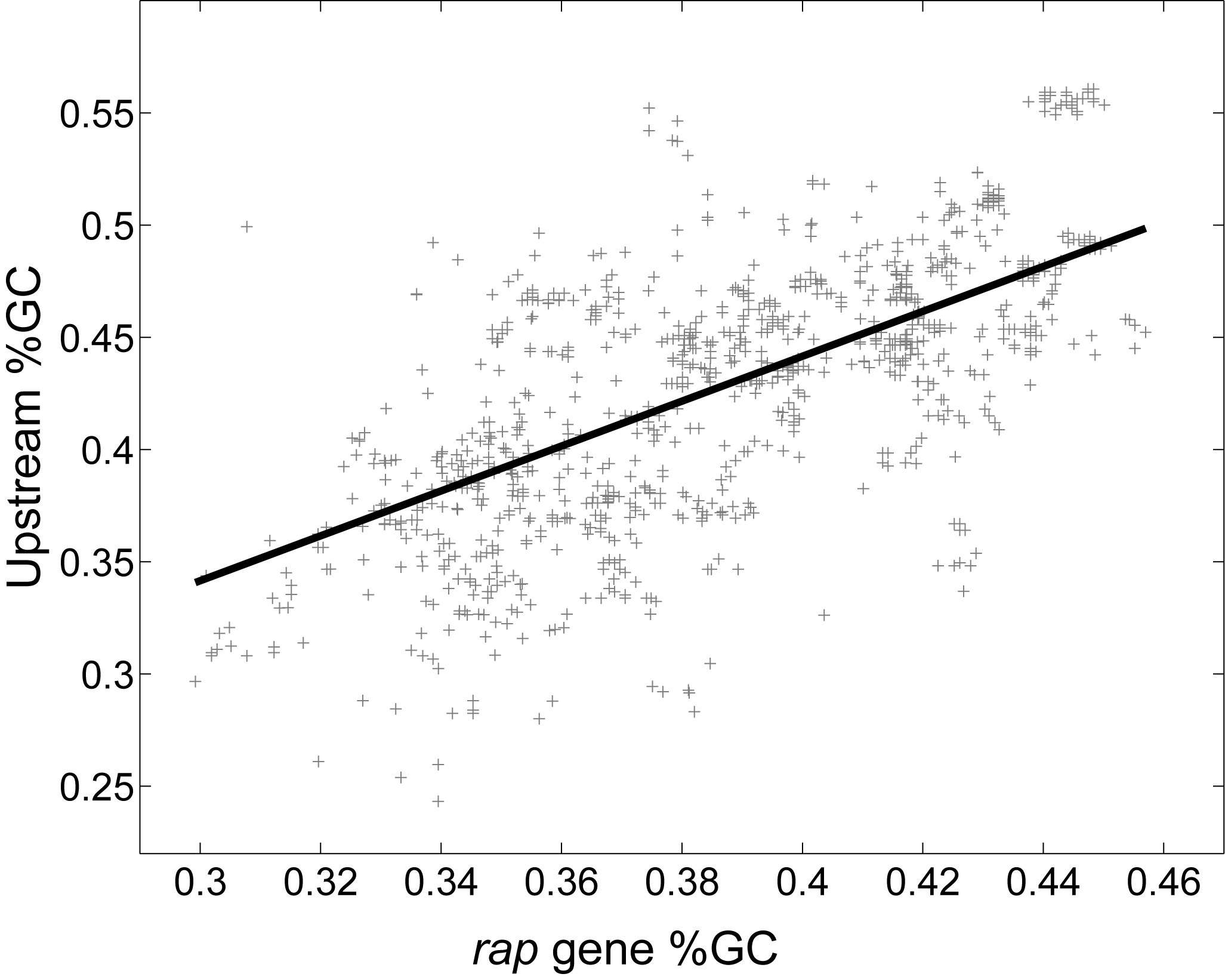

Supplement: S3 Fig — For each rap gene of the B. subtilis group, %GC content and the %GC content of the DNA sequence encoded from 1kbp to 300bp upstream to the rap start codon were calculated. A clear correlation was observed between the two %GC content measures (R2 = 0.69). Marked is the best-fit line (y = 1.1x). (TIF) [file pbio.2000330.s003.tif]

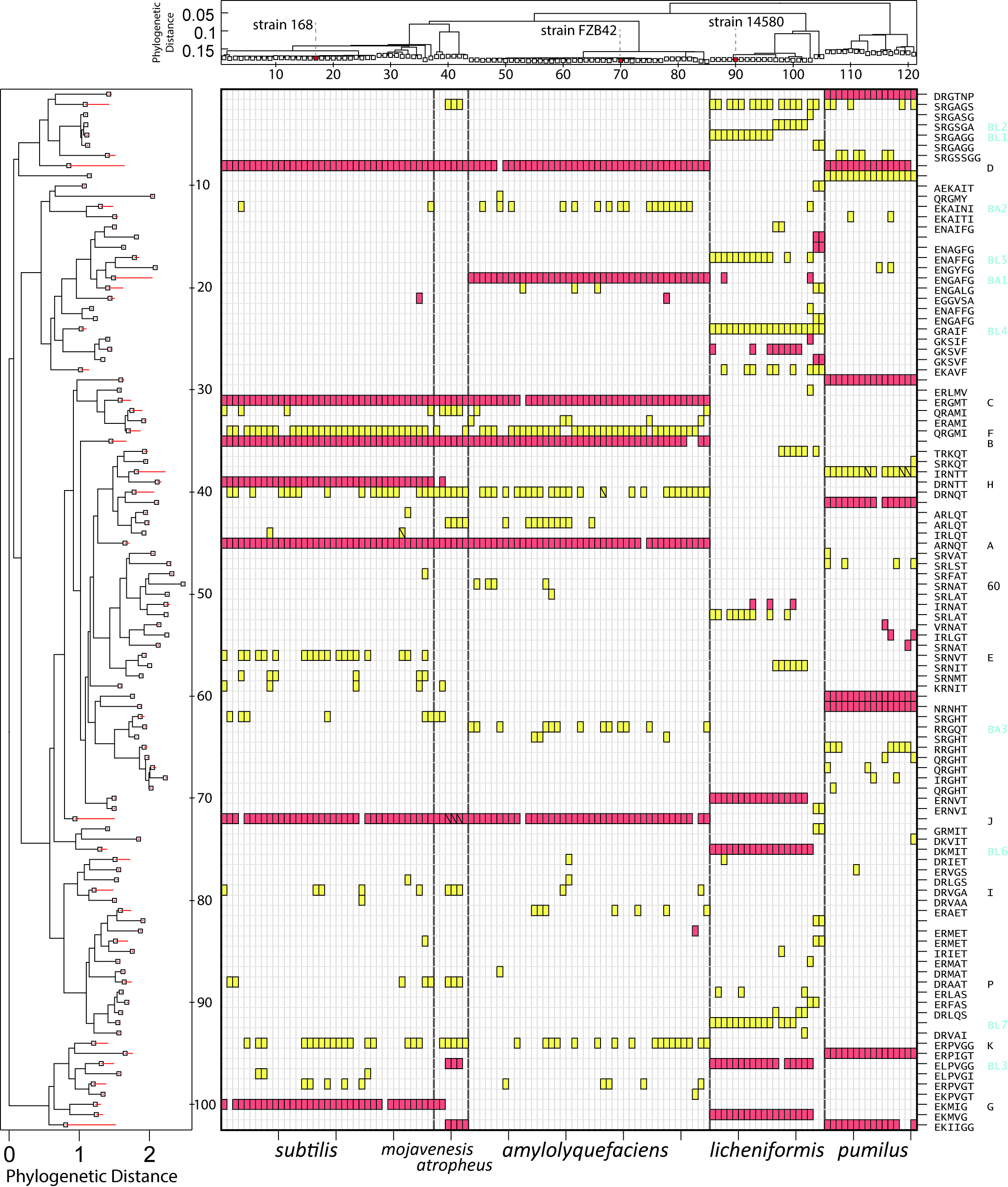

Supplement: S4 Fig — The presence of a Rap protein of a certain cluster in a given strain is marked by a colored rectangle, where the colors indicate whether the cluster is considered horizontally transferred (yellow) or fixed (red), based on the %GC content of the rap sequence (corresponding to background color of the cluster in Fig 2C). A diagonal line over the specific rectangle marks cases where two duplicates from the same cluster are found in a single genome (3 cases for orphan RapJ and 5 cases for Rap-Phr systems). Species are separated by thicker vertical lines and species names are marked below the matrix. The three strains which were used for experiments are specifically marked. Putative Phr autoinducers that define each cluster are marked to the right of the matrix. The Rap name is given as well. Names of newly characterized Rap proteins are given in cyan. Matrix column and row numbers are shown in intervals of 10. The relation between column number and strain info and between row number and cluster info are presented in S1 Data, tabs 3,4. (TIF) [file pbio.2000330.s004.tif]

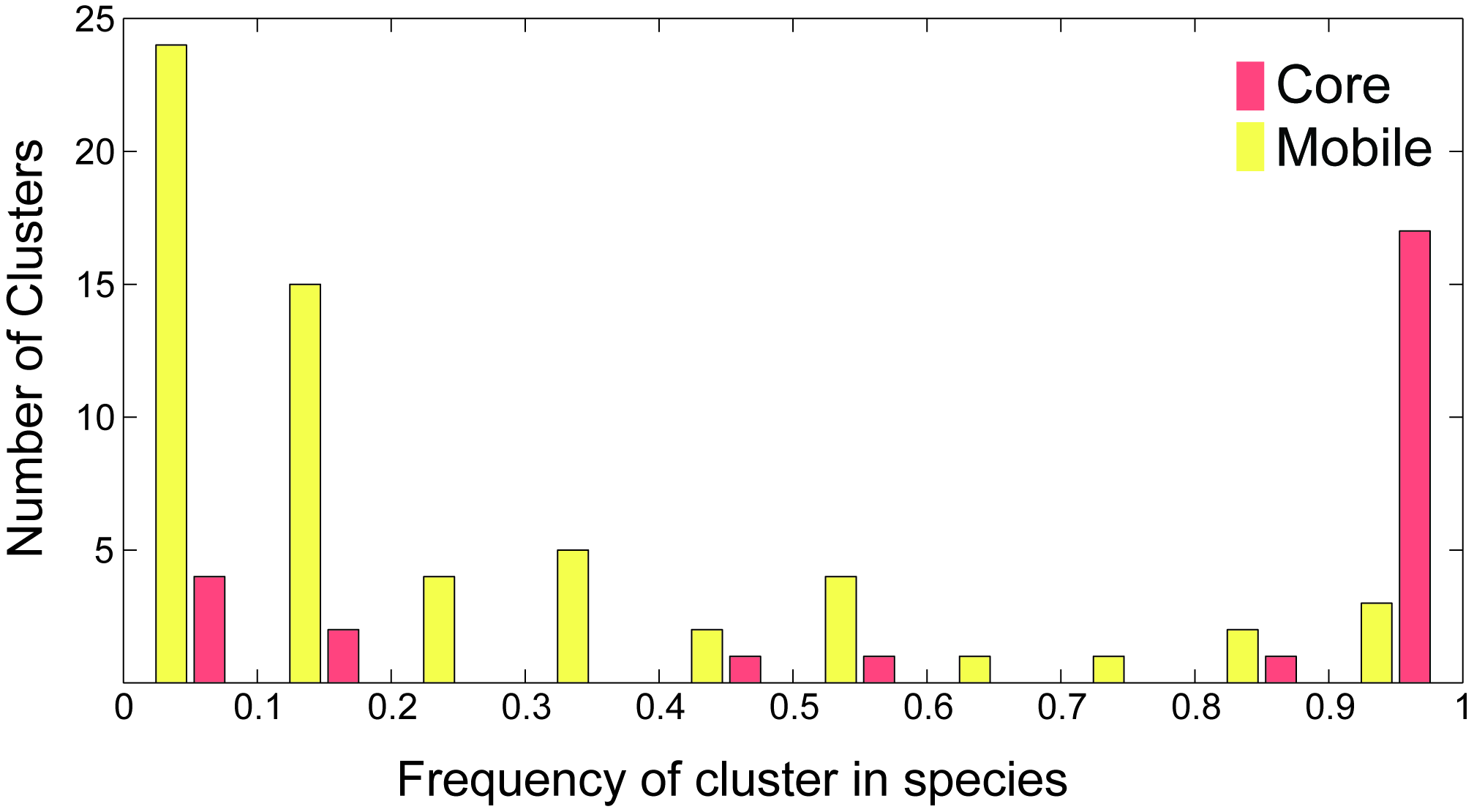

Supplement: S5 Fig — For each cluster present in the major species (B. subtilis, B. amyloliquefaciens, B. licheniformis and B. pumilus), the frequency of strains of each species where a rap gene belonging to this cluster exists was calculated (S5 Data). If a representative of a given cluster existed in more than one of the species, their frequency in the different species was averaged. Shown are the histograms of frequencies for clusters which were characterized as core (red) or as mobile (yellow), based on their %GC content. Clearly, mobile clusters mostly occur at low frequencies, while core clusters mostly occur at high frequencies. (TIF) [file pbio.2000330.s005.tif]

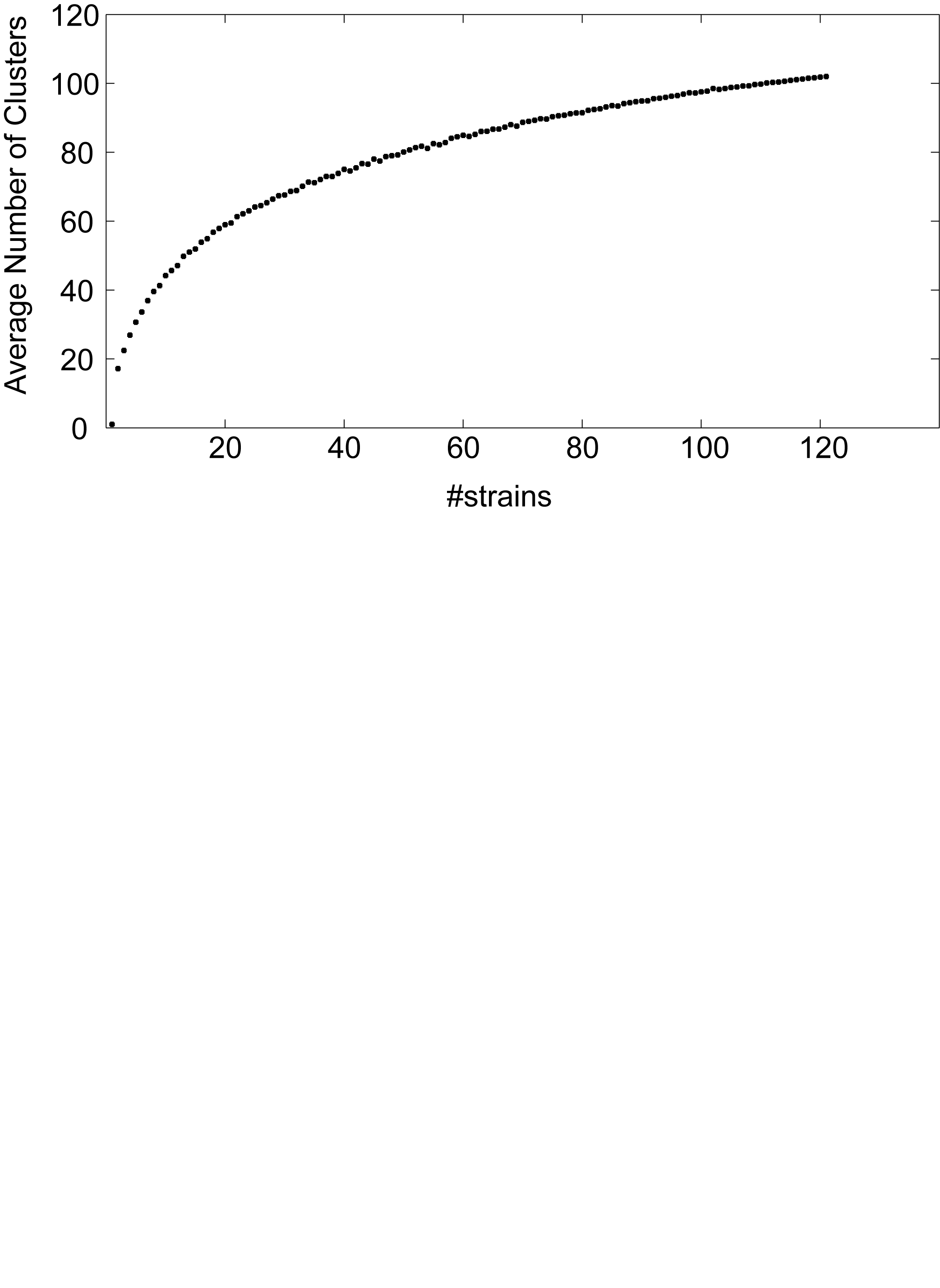

Supplement: S6 Fig — Shown are the average number of clusters identified as a function of the size of the group of strains examined. For each group size, 500 random samples were examined. The curve does not saturate even at maximal strain number. For the last 20 strains, the slope of newly identified clusters is ~0.2 clusters per new strain. (TIF) [file pbio.2000330.s006.tif]

A

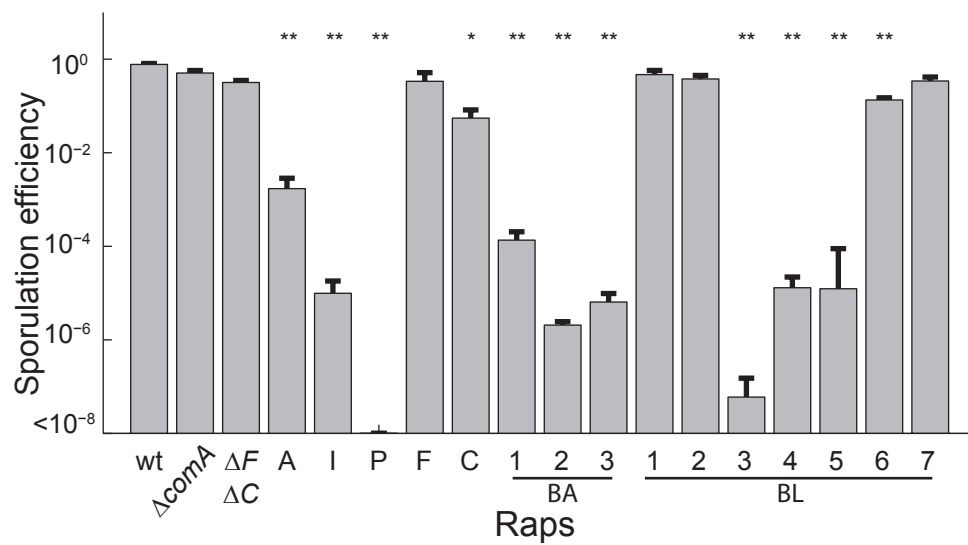

B

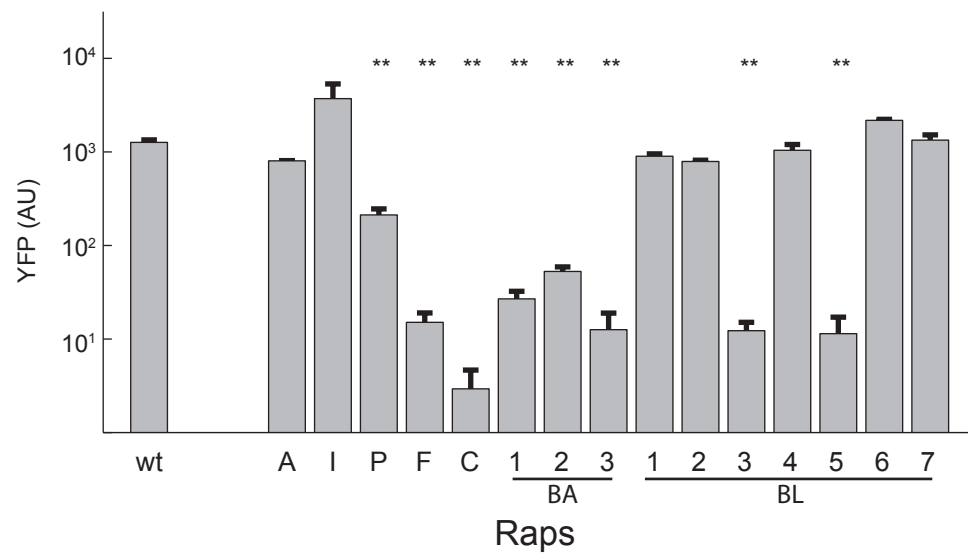

Supplement: S7 Fig — (A) Sporulation and (B) Psrf-YFP expression following expression of different Rap constructs (repeat of the data shown in Fig 3A of the main manuscript, S5 Data). Two additional controls, not shown in Fig 3A, are the sporulation efficiency of strains ΔcomA and ΔrapFphrF;ΔrapCphrC. A single asterisk indicates p<0.05, while two asterisks mark p<0.001 (two sample t-test between the strain and its parental background which lacks the Rap overexpression construct). (PDF) [file pbio.2000330.s007.pdf]

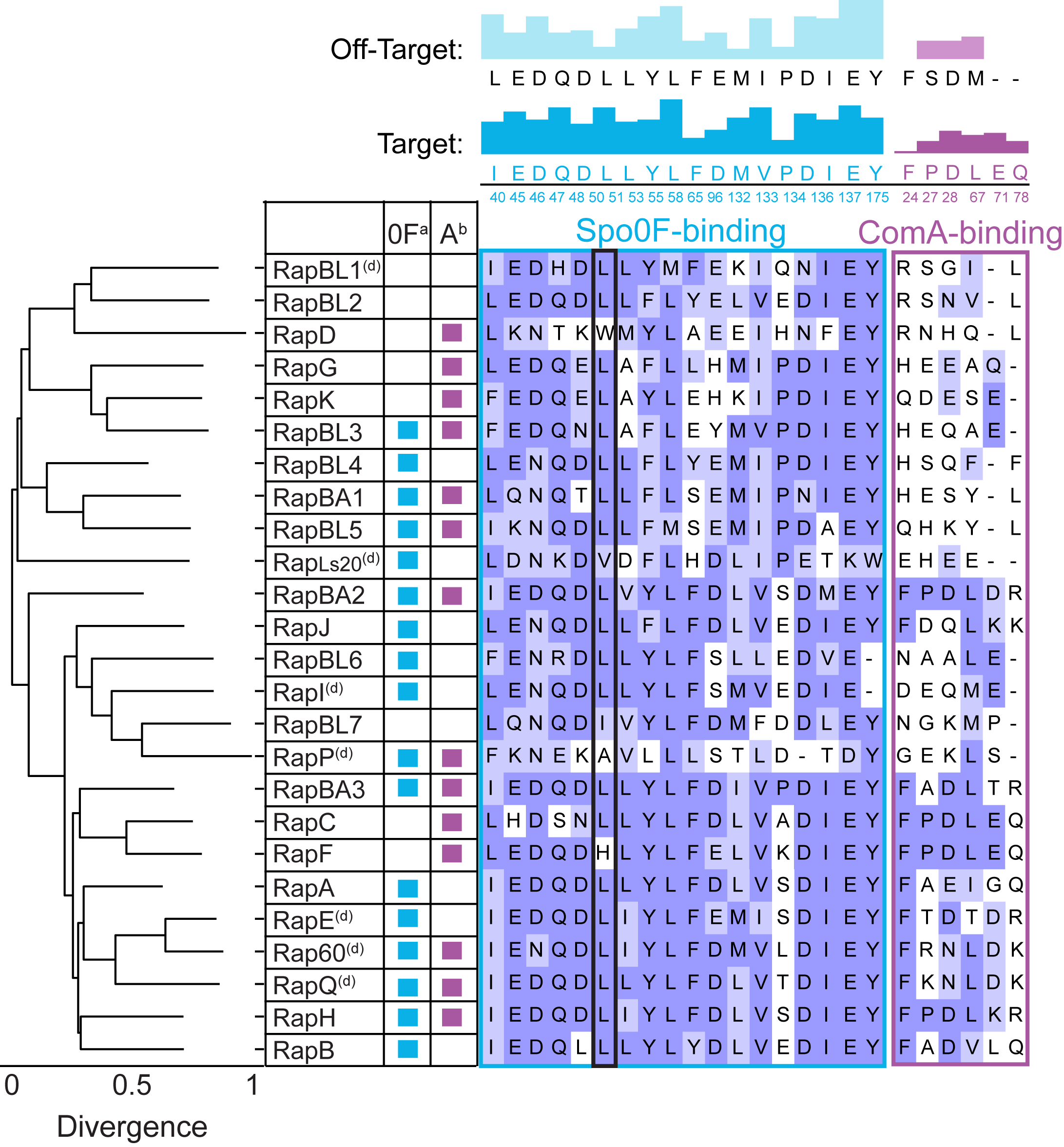

Supplement: S8 Fig — Sequence alignment of experimentally characterized Rap proteins at residues which were inferred to interact with Spo0F and ComA, based on the crystal structure of RapH in complex with Spo0F (green) [1] and of RapF in complex with the ComA DNA binding domain (red) [2]. Residue numbers and consensus are shown above the alignment. For each target, we calculated the consensus for sequences that affect the specific target (designated Target) and for sequences that do not affect it (designated OFF-Target). (TIF) [file pbio.2000330.s008.tif]

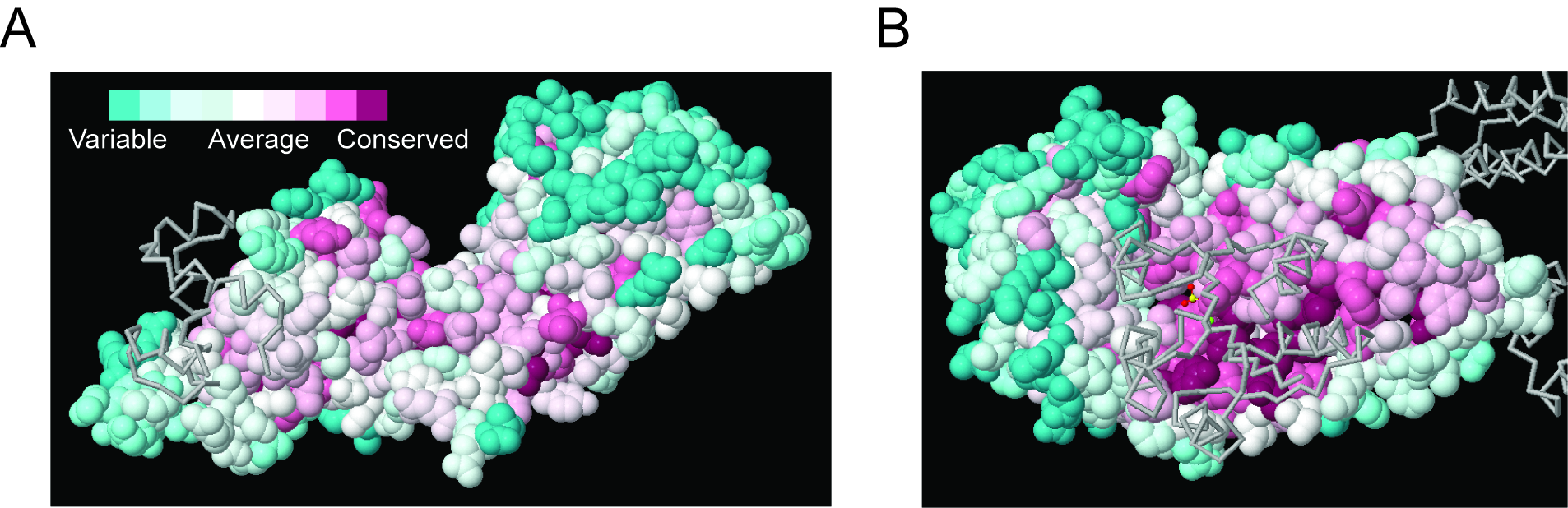

Supplement: S9 Fig — Output results of a Consurf analysis performed on the alignment of all B. subtilis group Rap proteins using the structure of (A) RapF in complex with the ComA DNA binding domain (PDB ID: 3ulq), or (B) RapH in complex with Spo0F (PDB ID: 3q15). ComA and Spo0F are shown in a stick representation at the left side (A), or the middle (B) of the respective Rap proteins. The Rap proteins are depicted using a space-filling view, where each residue is marked according to its level of conservation based on the legend shown in (A). Stick representation at the right of RapH in (B), corresponds to a RapH dimer appearing in the crystal structure. (TIF) [file pbio.2000330.s009.tif]

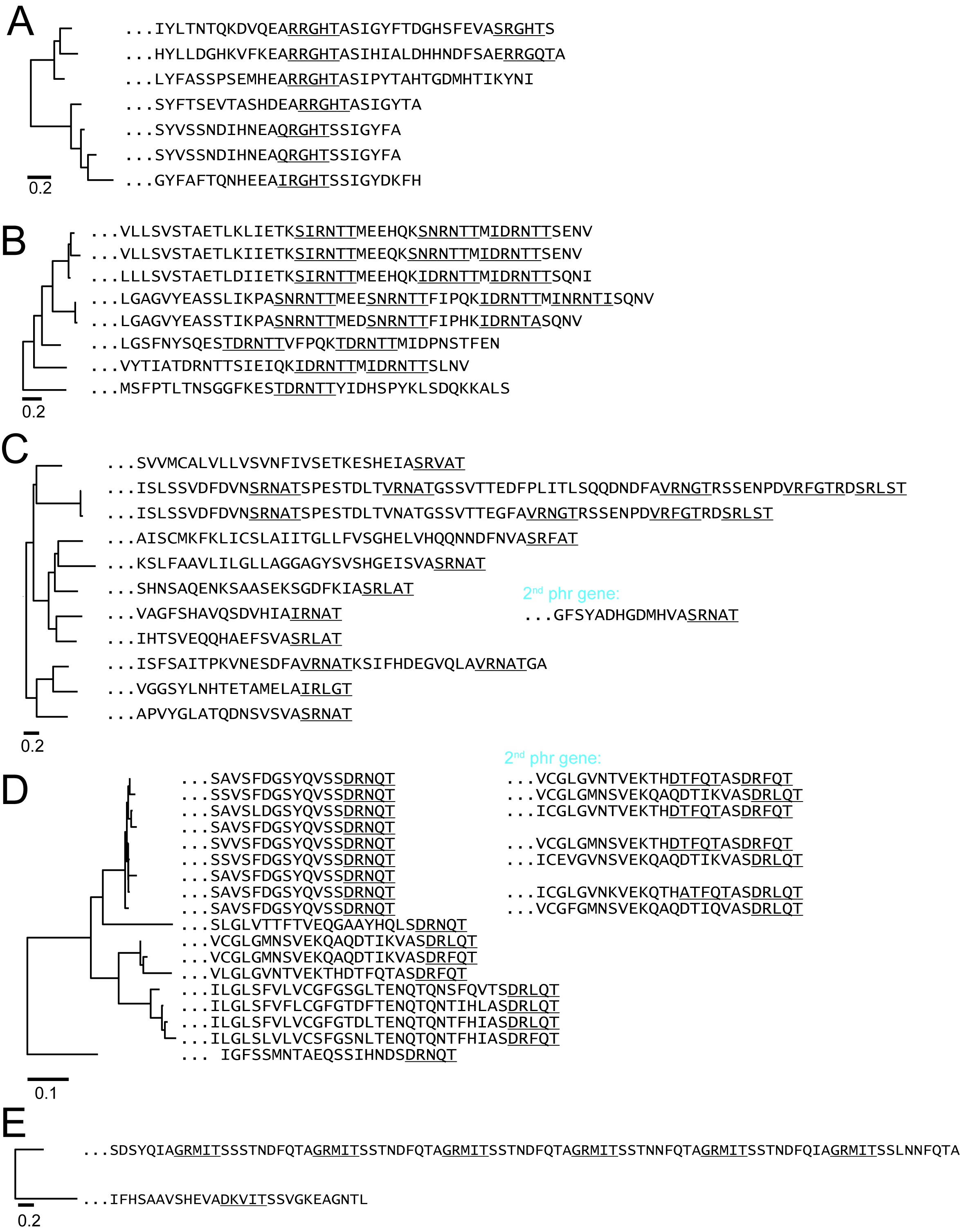

Supplement: S10 Fig — Shown are five more subtrees of the B. subtilis group Rap phylogeny, here some of the putative phr sequences encode multiple repeats of the autoinducer, with or without divergent amino acids. The subtree is based on the Rap amino acid sequence as presented in Fig 2C. Each leaf is marked with the sequence of the Phr prepeptide. Three dots indicate that the initial 20–30 amino acids (constituting the secretion signal sequence) are deleted. Putative autoinducer-like sequence are underlined. In (C,D) some of the rap genes are associated with two phr genes. In these cases, both sequences are shown. The scale bar marks the degree of amino acid divergence. (TIF) [file pbio.2000330.s010.tif]

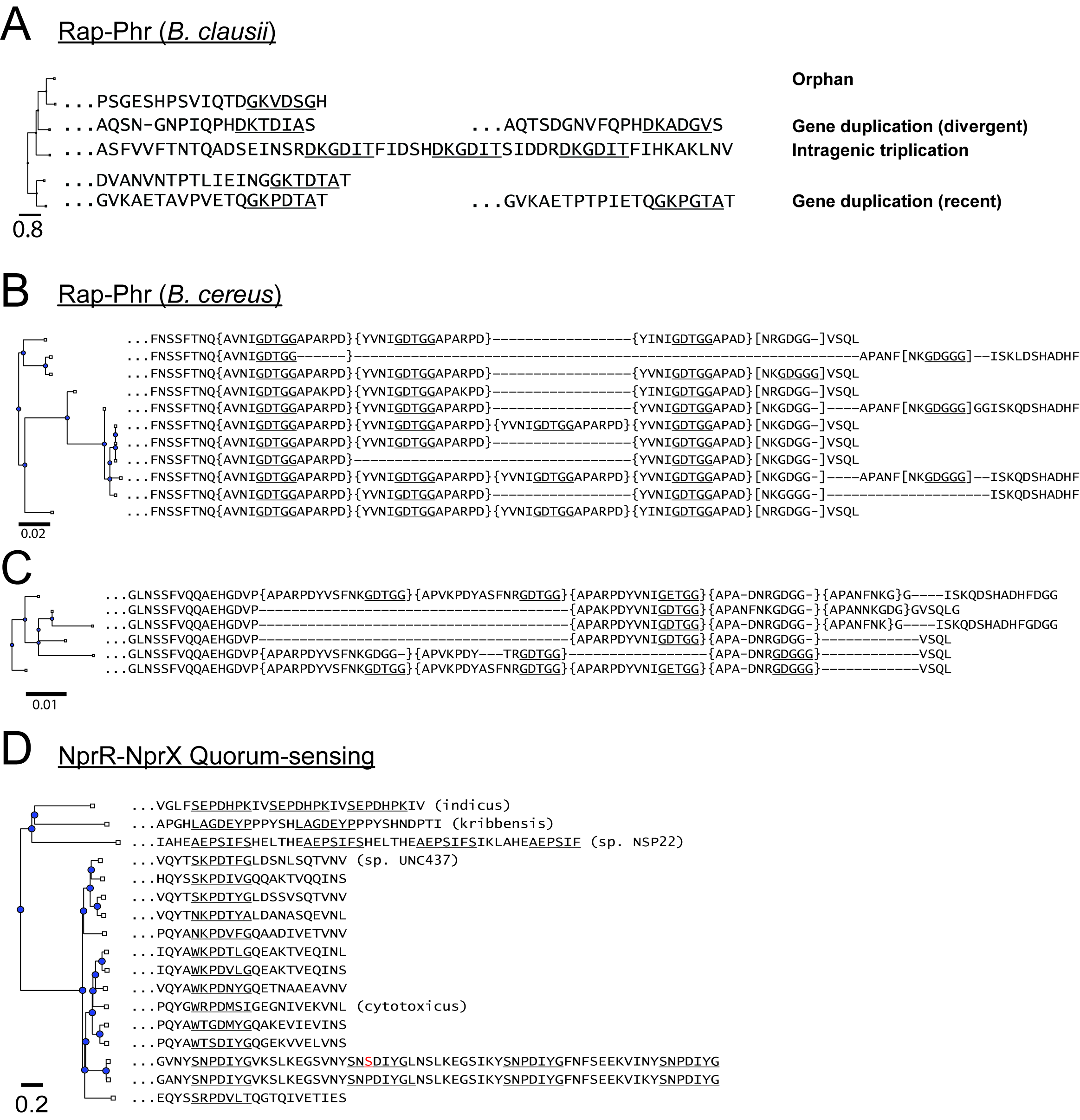

Supplement: S11 Fig — (A) Phylogenetic relation between the six Rap proteins encoded in the single fully sequenced B. clausii isolate. All of these Rap proteins are monophyletic within the B. subtilis Rap sub-tree (Fig 2B). The putative autoinducer sequence is underlined. (B,C) Two examples of rap sub-trees from B. cereus. The putative Phr autoinducer is underlined. Duplicated parts of the pre-peptide are shown with curly brackets. Putative autoinducer sequence identification was based on ref. [3]. Sequences are aligned according to the best match between duplicates in different species. Note the small divergence scale of the two systems. Putative Phr sequences are unknown for B. cereus and are therefore not marked. (D) Phylogenetic tree of NprR variants from B. cereus group and other species. If the species from which the Rap sequence was taken is not part of the B. cereus group sensu stricto, its name is marked in parentheses. NprX prepeptide sequence is shown and the putative autoinducer sequence is marked [4,5]. Three dots mark the absence of the secretion signal sequence from the pre-peptide sequence. (TIF) [file pbio.2000330.s011.tif]
