# Supplementary material for: Transient Duplication-Dependent Divergence and Horizontal Transfer Underlie the Evolutionary Dynamics of Bacterial Cell–Cell Signaling
Source: PLoS Biol. 2016 Dec 29;14(12):e2000330. doi: 10.1371/journal.pbio.2000330 (PMC5199041; doi:10.1371/journal.pbio.2000330)
Supplement: S1 Table — (DOCX) [file pbio.2000330.s012.docx]

**S1 Table**

| Rap Data#^1^ | Name | Accession | Strain | Putative Phr autoinducer Peptide | Note |
| --- | --- | --- | --- | --- | --- |
| 9 | BA1 | ABS76051 | *B. amy^a^.* | ENGAFG |  |
| 3 | BA2 | ABS72859 | *B. amy^a^.* | EKAINI |  |
| 5 | BA3 | ABS74335 | *B. amy^a^.* | RRGHT |  |
| 1484 | BL1 | AAU24735 | *B. lich^b^.* | SRGAGG |  |
| 1477 | BL2 | AAU22489 | *B. lich^b^.* | SRGAGS |  |
| 1476 | BL3 | AAU22336 | *B. lich^b^.* | ELPVGG |  |
| 1485 | BL4 | AAU24865 | *B. lich^b^.* | GRAIF |  |
| 1483 | BL5 | AAU24486 | *B. lich^b^.* | ENAFFG |  |
| 1479 | BL6 | AAU22627 | *B. lich^b^.* | DKMIT |  |
| 1486 | BL7 | AAU24904 | *B. lich^b^.* | - |  |
| 1761 | K_2_-RR | WP_016938737 | *B. siam^d^* | 5-mer: ERPVG  6-mer: ERPVGT |  |
| 2622 | K_2_-KK | WP_045510259  (Synthetically produced) | *B. amy^a^*. *strain CMW1* | 5-mer: EKPVG  6-mer: EKPVGT |  |
| 2168 | K_2_-KR | WP_019712648 | B. sub. ^c^ AUSI |  |  |
| 2013 | A |  | *B. subtilis^e^* | ARNQT | *phrA* deletion used |
| 2010 | C |  | *B. subtilis^e^* | ERGMT |  |
| 2018 | F |  | *B. subtilis^e^* | QRGMI |  |
| 2011 | I |  | *B. subtilis^e^* | DRVGA |  |
|  | P |  | *B. subtilis^e^* | DRAAT | RapP is a mutated form of *B. subtilis* strain 3610 RapP (Ben-dori et al, 2015) |

1. Index in the Rap database, S1,S2 Data files.
2. *Bacillus amyloliquefaciens* strain FZB42
3. *Bacillus licheniformis* strain ATCC 14580
4. *Bacillus subtilis* strain AUSI98
5. *Bacillus siamensis* strain KCTC 13613
