# Supplementary material for: Transient Duplication-Dependent Divergence and Horizontal Transfer Underlie the Evolutionary Dynamics of Bacterial Cell–Cell Signaling
Source: PLoS Biol. 2016 Dec 29;14(12):e2000330. doi: 10.1371/journal.pbio.2000330 (PMC5199041; doi:10.1371/journal.pbio.2000330)
Supplement: S2 Table — (DOCX) [file pbio.2000330.s013.docx]

**S3 Table – strain list**

| **strain name** | **genotype^a^** | **source reference^b^** |
| --- | --- | --- |
| AES101 | *B. subtilis* PY79 wild type | *Bacillus* genetic stock center |
| AES2010 | ∆*rapF-phrF*::*tet* | This study |
| AES1404 | *∆comA::Cm* | This study |
| AES1954 | ∆*rapC-phrC*:: *mls* | This study |
| AES2021 | *∆rapF-phrF:: tet*∆*rapC-phrC*:: *mls* | AES1954🡪AES2010 |
| AES2539 | *sacA:*:(P*_srf_*-3xYFP Cm) | AEC1003🡪AES101 |
| AES3140 | *sacA:*:(P*_spoIIG_*-3xYFP Cm) | AEC1103🡪AES101 |
| BS225 | *∆spo0A:: km* | kind gift from S. Ben Yehuda |
| AES2026 | *amyE*::(P*_srf_*-3xYFP Sp) | [[34](#_ENREF_34)] |
| AES2543 | ∆*rapF-phrF*:: tet *sacA*::(P*_srf_*-3xYFP Cm) | AES2539 🡪AES2010 |
| AES2249 | ∆*rapF-phrF*:: tet ∆*rapC-phrC*:: mls *amyE*::(*P_hs_-rapC*Sp) | AEC1954🡪AES2543 |
| AES2557 | ∆*rapF-phrF*:: tet *amyE*::(P_hs_-*rapF*Sp) *sacA*::(P*_srf_*-3xYFP Cm) | AEC954🡪AES2543 |
| AES2513 | ∆*rapF-phrF*:: tet ∆*rapC-phrC*:: mls *amyE*::(*P_hs_-rapC*Sp) sacA::(*P_srf_*-3xYFP Cm) | AEC958🡪AES2249 |
| AES2956 | *amyE*::(P_hs_-*rap1484*Sp) *sacA*::(P*_srf_*-3xYFP Cm) | AEC1102🡪AES2539 |
| AES2849 | *amyE*::(P_hs_-*rap1476*Sp) *sacA*::(P*_srf_*-3xYFP Cm) | AEC1069🡪AES2539 |
| AES2960 | *amyE*::(P_hs_-*rap1483*Sp) *sacA*::(P*_srf_*-3xYFP Cm) | AEC1071🡪AES2539 |
| AES2852 | *amyE*::(P_hs_-*rap1477*Sp) *sacA*::(P*_srf_*-3xYFP Cm) | AEC1070🡪AES2539 |
| AES3167 | *amyE*::(P_hs_-*rap1479*Sp) *sacA*::(P*_srf_*-3xYFP Cm) | AEC1128🡪AES2539 |
| AES2856 | *∆phrA:: tet* | this study |
| AES2853 | *amyE*::(P_hs_-*rap1485*Sp) *sacA*::(P*_srf_*-3xYFP Cm) | AEC1131🡪AES2539 |
| AES2958 | *amyE*::(P_hs_-*rap3*Sp) *sacA*::(P*_srf_*-3xYFP Cm) | AEC1101🡪AES2539 |
| AES2855 | *amyE*::(P_hs_-*rap5*Sp) *sacA*::(P*_srf_*-3xYFP Cm) | AEC1129🡪AES2539 |
| AES2854 | *amyE*::(P_hs_-*rap9*Sp) *sacA*::(P*_srf_*-3xYFP Cm) | AEC1068🡪AES2539 |
| AES2850 | *amyE*::(P_hs_-*rap1486*Sp) *sacA*::(P*_srf_*-3xYFP Cm) | [[34](#_ENREF_34)] |
| AES1378 | *amyE*::(PrapP-rapPN236T Sp) | AEC1102🡪AES2539 |
| AES2949 | *amyE*::(P_hs_-*rapI* Sp) sacA::(Psrf-3xYFP Cm) | AEC1130🡪AES2539 |
| AES2910 | *∆spo0A::kan sacA*::(P_srf_-3xYFP Cm) | BS225 🡪AES2539 |
| AES3615 | *∆spo0A::kan amyE*::(Prap-rapPN236T Sp) *sacA*::(P_srf_-3xYFP Cm) | AES1378 🡪AES2910 |
| AES3049 | *∆spo0A::kan amyE*::(P_hs_-*rap1484*Sp) *sacA*::(P_srf_-3xYFP Cm) | AEC1102 🡪AES2910 |
| AES2922 | *∆spo0A::kan amyE*::(P_hs_-*rap1476*Sp) *sacA*::(P_srf_-3xYFP Cm) | AEC1069 🡪AES2910 |
| AES2954 | *∆spo0A::kan amyE*::(P_hs_-*rap1483*Sp) *sacA*::(P_srf_-3xYFP Cm) | AEC1071 🡪AES2910 |
| AES2926 | *∆spo0A::kan amyE*::(P_hs_-*rap1477*Sp) *sacA*::(P_srf_-3xYFP Cm) | AEC1070 🡪AES2910 |
| AES2953 | ∆spo0A::kan *amyE*::(P_hs_-*rap1479*Sp) *sacA*::(P_srf_-3xYFP Cm) | AEC1128 🡪AES2910 |
| AES3049 | ∆spo0A::kan *∆phrA:: tet sacA*::(P_srf_-3xYFP Cm) | AES2856🡪AES2910 |
| AES2934 | *∆spo0A::kan* ∆*rapF-phrF*:: tet *amyE*::(P_hs_-*rapF*Sp) *sacA*::(P_srf_-3xYFP Cm) | AES2910 🡪AES2557 |
| AES2932 | *∆spo0A::kan* ∆*rapF-phrF*:: tet ∆*rapC-phrC*:: mls *amyE*::(*P_hs_-rapC*Sp) sacA::(*P_srf_*-3xYFP Cm) | AES2910 🡪 AES2449 |
| AES2928 | *∆spo0A::kan amyE*::(P_hs_-*rap1485* Sp) *sacA*::(P_srf_-3xYFP Cm) | AEC1131 🡪AES2910 |
| AES3027 | *∆spo0A::kan amyE*::(P_hs_-*rap3* Sp) *sacA*::(P_srf_-3xYFP Cm) | AEC1101 🡪AES2910 |
| AES2942 | *∆spo0A::kan amyE*::(P_hs_-*rap5* Sp) *sacA*::(P_srf_-3xYFP Cm) | AEC1129 🡪AES2910 |
| AES2930 | *∆spo0A::kan amyE*::(P_hs_-*rap9* Sp) *sacA*::(P_srf_-3xYFP Cm) | AEC1068 🡪AES2910 |
| AES2924 | *∆spo0A::kan amyE*::(P_hs_-*rap1486* Sp) *sacA*::(P_srf_-3xYFP Cm) | AEC1072 🡪AES2910 |
| AES2938 | *∆rapK-phrK:: tet* | this study |
| AES3473 | *∆rapK-phrK:: tet sacA*::(P*_spoIIG_*-3xYFP Cm) | AEC1103🡪AES2938 |
| AES3467 | *∆rapK-phrK::tet amyE*::(P_hs_-*rap1761*Sp) *sacA*::(P*_spoIIG_*-3xYFP Cm) | AEC1123🡪AES3473 |
| AES3520 | *∆rapK-phrK::tet amyE*::(P_hs_-*rap2168*Sp) sacA::(P*_spoIIG_*-3xYFP Cm) | AEC1126🡪AES3473 |
| AES3147 | *∆phrA:: tet sacA*::(P*_spoIIG_*-3xYFP Cm) | AEC1103🡪AES2856 |
| AES3145 | *amyE*::(P_hs_-*rapI*Sp) sacA::(P*_spoIIG_*-3xYFP Cm) | AEC1130🡪AES3140 |
| AES3151 | *amyE*::(P_hs_-*rap9*Sp) sacA::(P*_spoIIG_*-3xYFP Cm) | AEC1068🡪AES3140 |
| AES4228 | *∆spo0A::kan amyE*::(P_hs_-*rapJ*Sp) *sacA*::(P_srf_-3xYFP Cm) | AEC1255🡪AES2910 |
| AES4230 | *∆spo0A::kan amyE*::(P_hs_-*rapD*Sp) *sacA*::(P_srf_-3xYFP Cm) | AEC1256🡪AES2910 |
| AES4232 | *∆spo0A::kan amyE*::(P_hs_-*rapB*Sp) *sacA*::(P_srf_-3xYFP Cm) | AEC1257🡪AES2910 |
| AES4234 | *amyE*::(P_hs_-*rapJ*Sp) sacA::(P*_spoIIG_*-3xYFP Cm) | AEC1255🡪AES3140 |
| AES4236 | *amyE*::(P_hs_-*rapD*Sp) sacA::(P*_spoIIG_*-3xYFP Cm) | AEC1256🡪AES3140 |
| AES4242 | *amyE*::(P_hs_-*rapB*Sp) sacA::(P*_spoIIG_*-3xYFP Cm) | AEC1257🡪AES3140 |
| AEC310 | ece174 | [[79](#_ENREF_79)] |
| AEC777 | pDR111 | [[44](#_ENREF_44)] |
| AEC954 | pDR111-P_hs_*RapF* | this study |
| AEC958 | pDR111-P_hs_*RapC* | this study |
| AEC945 | pDL30-P*_srf_*-3xYFP | [[34](#_ENREF_34)] |
| AEC1003 | ece174-P*_srf_*-3xYFP | this study |
| AEC1103 | ece174-P*_spoIIG_*-3xYFP | this study |
| AEC1069 | pDR111-P_hs_*Rap1476* | this study |
| AEC1071 | pDR111-P_hs_*Rap1483* | this study |
| AEC1070 | pDR111-P_hs_*Rap1477* | this study |
| AEC1130 | pDR111-P_hs_*RapI* | this study |
| AEC1101 | pDR111-P_hs_*Rap3* | this study |
| AEC1129 | pDR111-P_hs_*Rap5* | this study |
| AEC1131 | pDR111-P_hs_*Rap1485* | this study |
| AEC1128 | pDR111-P_hs_*Rap1479* | this study |
| AEC1068 | pDR111-P_hs_*Rap9* | this study |
| AEC735 | ece174 P*_rapP_ rapP*^N236T^ | [[34](#_ENREF_34)] |
| AEC1102 | pDR111-P_hs_*Rap1484* | this study |
| AEC1072 | pDR111-P_hs_*Rap1486* | this study |
| AEC1121 | pDR111-P_hs_*RapK* | this study |
| AEC1123 | pDR111-P_hs_*Rap1761* | this study |
| AEC1126 | pDR111-P_hs_*Rap2168* | this study |
| AEC1255 | pDR111-P_hs_*RapJ* | this study |
| AEC1256 | pDR111-P_hs_*RapD* | this study |
| AEC1257 | pDR111-P_hs_*RapB* | this study |

1. All B. subtilis strains used in this work are of a PY79 background. All plasmids are kept in an E. coli DH12 background.
2. A 🡪 mark indicates the transformation of genetic material from the source strain (left) to the receiver strain (right), resulting in the indicated genotype.
