# Supplementary material for: Transient Duplication-Dependent Divergence and Horizontal Transfer Underlie the Evolutionary Dynamics of Bacterial Cell–Cell Signaling
Source: PLoS Biol. 2016 Dec 29;14(12):e2000330. doi: 10.1371/journal.pbio.2000330 (PMC5199041; doi:10.1371/journal.pbio.2000330)
Supplement: S3 Table — (DOCX) [file pbio.2000330.s014.docx]

**S4 Table – primer list**

| **Primer name** | **Primer sequence^a^** |
| --- | --- |
| hsRap2168-F | ggcgctagcagaattttgacggaaagagg |
| hsRap2168-R | gccgcatgctcatcctattccctccaact |
| hsRap1761-F | ccggtcgacgaaacgggaaattgcgtgat |
| hsRap1761-R | ccggctagcttataatccctccaggtgcc |
| hsRap9-F | cgggctagccacaaaatgggaaagggagg |
| hsRap9-R | ggcgcatgctttacatgttttcattacga |
| hsRap5-F | cgggctagccagacgaaatgctaggaggg |
| hsRap5-R | ggcgcatgcttaaatttcataaagacaatcc |
| hsRap3-F | cgggctagcagtgattagagggaggggta |
| hsRap3-R | ggcgcatgctcatgatatagcctccgtaa |
| hsRap1486-F | gccgctagctgcgagaaaggaagaggata |
| hsRap1486-R | ggcgcatgctcactttaactcctccgttc |
| hsRap1485-F | gccgctagctgacatgggaagaaaggaag |
| hsRap1485-R | ggcgcatgctcactttaactcctccgttc |
| hsRap1484-F | cttgctagccaaagattggagggaaaaga |
| hsRap1484-R | gctgcatgccctattctcctcctcttccg |
| hsRap1483-F | gccgctagcatacgaaaggaatggtggga |
| hsRap1483-R | cgggcatgctcatgagacaagttcctccg |
| hsRap1479-F | gttgtcgactgtacgaagtcttgggaggc |
| hsRap1479-R | ggcgcatgcttagctttcatacaacgcat |
| hsRap1477-F | ctggctagcagtaaagtgtgggaggtttg |
| hsRap1477-R | tccgcatgcttatgtggaatatttgtaattat |
| hsRap1476-F | cggaagctttcttaaaaagaaagaggggg |
| hsRap1476-R | cctgctagctcatgacaatacctcactcc |
| hsRapF-F | gatttaagtcgacttatgaagggagggatttgcaaacgtgacaggtgtcatatcttcttcttcc |
| hsRapF-R | taatagctagcttagacttcaatttcatacaaactcactcctcct |
| hsRapC-F | gatataagtcgacgatgggagagggtgaaggaatgaagagtggggtaattcc |
| hsRapC-R | tatagctagcttagatttcaatttcatacaaaccttcactcc |
| hsRapJ-F | ccggctagctagcaggaggaaggaggcagaccg |
| hsRapJ-R | ccggcatgcctattgaaaacgctgctcggcaag |
| hsRapB-F | ccggctagcccaattagaaaatagaaagggttgg |
| hsRapB-R | cggcatgctcatacttcatataaacaatcgttcc |
| hsRapD-F | ccggctagcccaatagcaaagaggtaggaaaatg |
| hsRapD-R | cgggcatgctcagccgcgcatgataaaaaaagcggc |
| RapK-P1 | ctgctgctcctgtcttggca |
| RapK-P2 | tgcccgcagctgtgacaacggctctcctcgtgaagaatgcagc |
| RapK-P3 | gtagcgcggtggtcccacgccctgctctgtggagcg |
| RapK-P4 | tcggctgtagcaatggggct |
| RapF-P1 | atcgaggcggctgatcc |
| RapF-P2 | ccggaggtgtagcatgtctcattcaattttgagggttcgttacaccgcatgtttgga |
| RapF-P3 | gcctattttttgtgaatcgattatgtcttttgcgtccatcggcggttttttcg |
| RapF-P4 | tcggaccgcacaatgtgt |
| RapC-P1 | agtggcttccggcgatt |
| RapC-P2 | ctatgagtcgcttttgtaaatttggaaagttataggccgtcccaaccc |
| RapC-P3 | ctcggatcccatttccccctttgatttttatgccgccttcatggaga |
| RapC-P4 | cggttttggccgcagag |
| ComA-P1 | aagttggaccggactggaat |
| ComA-P2 | ttttctaatgtcactaacctgccaaactgttcgctcggttcag |
| ComA-P3 | agtaatccgcccgacggtatagcggtccattgaatacagc |
| ComA-P4 | ggtgagccggtgatgtttac |
| phrA-P1 | agaggacacgtcgcagct |
| phrA-P2 | gtagcgcggtggtcccacccgcaacgagcaacaaacct |
| phrA-P3 | tgcccgcacgtgtgacaatgcataaaaaaagacccttagggcttttt |
| phrA-P4 | cacactggccccgtgtga |
| Psrf-sacA-F | atggggaattcgttgtaagacgctc |
| Psrf-sacA-R | cgcggatccttaaagctttttatacag |
| PspoIIG-F | ggcgaattcgtggaaaaaaagctgccgtc |
| PspoIIG-R | cgtgctagcagcttcccagccggatgttc |

1. An underline marks a restriction cut site.
